# Scale up of a *Plasmodium falciparum* elimination program and surveillance system in Kayin State, Myanmar

**DOI:** 10.12688/wellcomeopenres.12741.2

**Published:** 2017-12-22

**Authors:** Daniel M. Parker, Jordi Landier, Aung Myint Thu, Khin Maung Lwin, Gilles Delmas, François H. Nosten, The Malaria Elimination Task Force Group

**Affiliations:** 1 Shoklo Malaria Research Unit, Mahidol-Oxford Tropical Medicine Research Unit, Faculty of Tropical Medicine, Mahidol University, Mae Sot, Thailand; 2 Centre for Tropical Medicine and Global Health, Nuffield Department of Medicine, University of Oxford, Oxford, OX3 7BN, UK

**Keywords:** Myanmar, Kayin, Karen, Burma, Plasmodium falciparum, elimination, mass drug administration, early diagnosis and treatment, operational research

## Abstract

**Background:** Myanmar has one of the largest malaria burdens in the Greater Mekong Subregion (GMS). Throughout the GMS, *Plasmodium falciparum* parasites are increasingly resistant to artemisinin combination therapies. Given that there are no current alternative treatment therapies, one proposed solution to the threat of untreatable *P. falciparum* malaria is to eliminate the parasite from the region. Several small-scale elimination projects have been piloted in the GMS, including along the Myanmar-Thailand border. Following the success of the pilot elimination project along the Myanmar-Thailand border, there was a scale up to a broad area of Eastern Kayin State, Myanmar. Here we describe the establishment of the scale up elimination project in Easter Kayin State.

**Methods:** The scale up relied on geographic reconnaissance and a geographic information system, community engagement, generalized access to community-based early diagnosis and treatment, near real-time epidemiological surveillance, cross sectional malaria prevalence surveys and targeted mass drug administration in villages with high prevalence of *P. falciparum* malaria. Molecular markers of drug resistance were also monitored in individuals with symptomatic and asymptomatic infections.

**Discussion:** This protocol illustrates the establishment of an elimination project and operational research in a remote, rural area encompassing several armed groups, multiple political organizations and a near-absent health care infrastructure. The establishment of the project relied on a strong rapport with the target community, on-the-ground knowledge (through geographic surveys and community engagement), rapid decision making and an approach that was flexible enough to quickly adapt to a complex landscape. The elimination project is ongoing, now over three years in operation, and assessment of the impact of this operational research will follow. This project has relevance not only for other malaria elimination projects but also for operational research aimed at eliminating other diseases.

## Abbreviations

ACT: artemisinin combination therapy; CE: community engagement; MDA: mass drug administration; MP: malaria post; MPW: malaria post worker; GIS: geographic information system; qPCR: quantitative PCR; RDT: rapid diagnostic test

## Introduction

Malaria is endemic in Myanmar and the Greater Mekong Subregion (GMS) and it is a major cause of morbidity. The main Plasmodial species involved are *Plasmodium falciparum* and *Plasmodium vivax*. Transmission is seasonal and caused by multiple diverse Anopheles vectors ^1– 3^. In several parts of the GMS *P. falciparum* has become resistant to almost all available antimalarials, including artemisinin, artemisinin derivatives and partner drugs ^4^. There are recent reports of a single dihydroartemisinin-piperaquine resistant strain that first emerged on the Thailand-Cambodian border (in Pailin Province) has now spread to southern Vietnam, after previously spreading into northeastern Thailand and southern Laos ^5^. The emergence and subsequent spread of drug and multi-drug resistant *P. falciparum* malaria presents a major threat to the region and the rest of the world. Given the paucity of new drugs, one proposed solution is to attempt elimination of *P. falciparum* malaria before complete resistance to antimalarials is widespread. Several projects throughout the region have been established in order to test the feasibility of targeted *P. falciparum* elimination, including projects in Vietnam, Cambodia, Laos and Myanmar ( https://clinicaltrials.gov/ct2/show/NCT01872702). Most rely on a combination of public health interventions, usually based on community engagement, early diagnosis and treatment and targeted mass drug administration (MDA).

Between 2012 and 2014, a series of malaria prevalence surveys were conducted on the Thailand-Myanmar border using a highly sensitive quantitative PCR (qPCR) approach ^6, 7^. Several villages with high prevalence of subclinical infections were chosen for pilot elimination work using a combination of village health workers, community engagement (CE) and MDA, including dihydroartemisinin-piperaquine with a single low dose of primaquine ^6^. In four study villages on the Myanmar side of the border, the safety and acceptability of this intervention were carefully evaluated and the impact was measured through repeated (three monthly) mass blood screenings ^8– 10^. Detailed entomological evaluations were also conducted throughout the 24 months of the pilot study ^11^. The results indicated that the strategy is safe and effective in rapidly eliminating the sub-microscopic reservoir of malaria parasites and in reducing transmission to mosquito vectors ^12^.

These encouraging results motivated a scale up of the elimination project to much of Eastern Kayin State, Myanmar. Here we describe the logistics and establishment of this malaria elimination program in Eastern Kayin State, Myanmar from 2014 through 2017. The impact of the interventions are monitored through the analysis of observational data collected through longitudinal passive case detection at community-based malaria diagnosis and treatment centers (malaria posts (MPs)) and through cross-sectional blood screenings conducted in targeted villages. This protocol helps to fill an important gap in the literature on operational research programs aimed at eliminating malaria ^13– 15^.

## Methods

**Figure 1.**
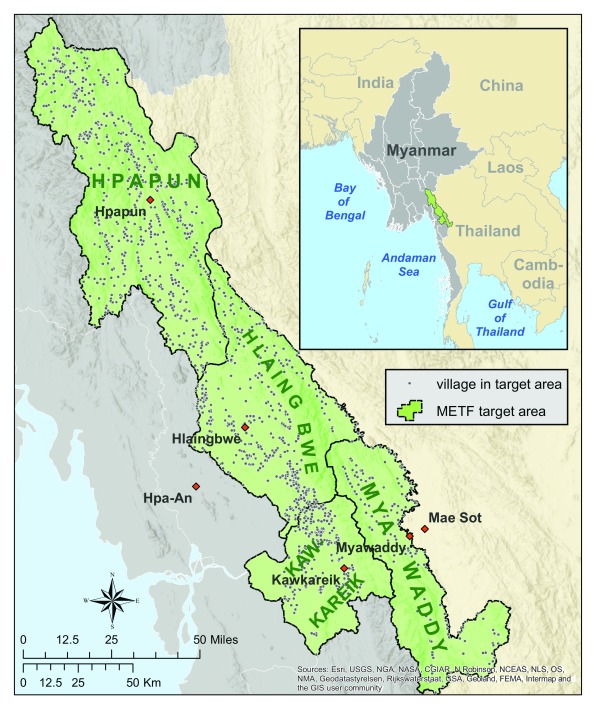
Map of target area and villages within the target area. METF, Malaria Elimination Task Force.

The target area consists of four townships of Kayin State, Myanmar: Myawaddy, Kawkareik, Hlaingbwe and Hpapun ( Figure 1). Much of the area has been in civil conflict for over half a century, with no accurate census or map ^16^ since World War II. In order to establish an operational malaria elimination project, it was therefore crucial to first develop an understanding of the geography and demography and to engage with the communities.

**Figure 2.**
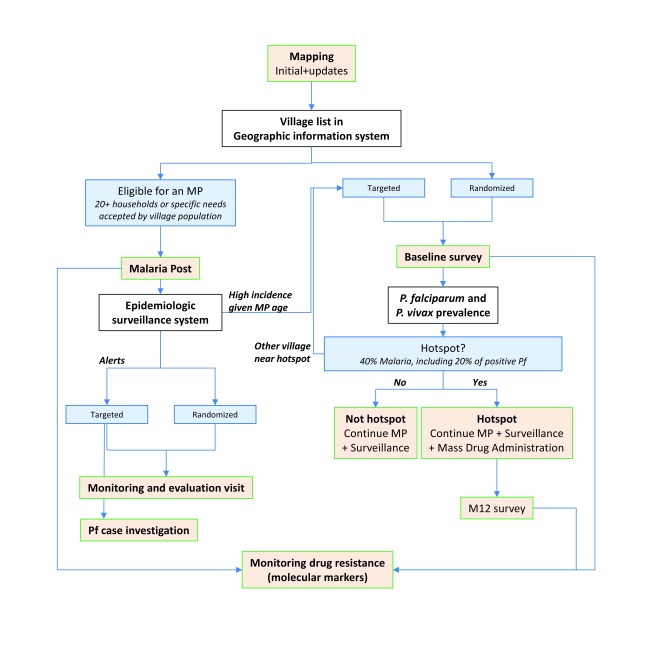
Flow-chart of the different components of Malaria Elimination Task Force elimination strategy and of the allocation of village-level interventions. MP, Malaria Post.

These reconnaissance efforts were then followed up by the establishment of a dense network of MPs, epidemiological and drug resistance surveillance systems, surveys for measuring prevalences of malaria in villages, and targeted MDA in cases where high prevalences of malaria were identified ( Figure 2).

### Mapping and geographic information system (GIS)

In order to understand the settlement demography and geography of the region, the area was systematically mapped using field teams and satellite-enabled geo-referencing devices. The devices simultaneously used GPS (global position system) and GLONAS (Globalnaya navigatsionnaya sputnikovaya sistema) satellites to increase accuracy of geographical coordinates (latitude and longitude). Mapping teams were composed of community members familiar with the region who were trained in the field by an experienced geographer and assistants. Politically sensitive areas and conflict zones were first approached with CE experts to gain local and regional support for the mapping activities.

From December of 2013 to December 2016, three waves of mapping were conducted. The first survey focused on whether or not malaria services existed in a community, whether or not they were properly staffed and stocked, names of villages, and the number of houses in a village. A second wave of surveys (conducted in 2014) aimed to correct any missing geographic points that were missing from the first wave, to fill in any gaps in the target area map, and to identify the locations of referral clinics. In 2015–2016, a third wave of mapping and surveys included a small set of economic indicators, including basic questions about agricultural development, transportation capabilities, electricity and water sources.

Data from the forms were entered into spread sheets and merged with geographic references downloaded from each GPS unit. These data formed the basic architecture for the project’s GIS. R statistical software (version 3.1.0) and Python programming language were used for data tabulations and merging; mapping of the data was primarily done using ArcGIS10.2, and QGIS 2.4 was used for creating and manipulating spatial shape files. The core GIS data were stored in a file geodatabase (file type .gdb). Each mapped village was assigned an arbitrary numeric identification code and all information relating to a village (reports, samples, logistics) was labelled using this identification code.

### Community engagement (CE)

The malaria elimination project relied on widespread participation and cooperation within and between communities ^9^. The project was therefore heavily dependent on the ability to properly engage with target communities. A CE team was formed at the beginning of the project and team members helped facilitate all aspects of the project.

The CE team created and distributed community engagement materials in order to sensitize and explain the project to villagers. Materials included posters and audio announcements (statements that were created by the CE team but given to village headmen to announce in periodic village announcements). METF posters, in S’kaw Karen and Burmese languages, that encourage people to visit malaria posts when they feel sick were placed in malaria posts (MPs) throughout the target area.

MPs were typically established in batches. Prior to malaria post worker (MPW) training, the CE team asked for a meeting with local health workers, village headmen, and other leaders. During this initial meeting the elimination program, MP system and CE were all explained to local leaders. Prior to malaria prevalence surveys the CE team met with township-level health care leaders and village headmen who were asked for permission to conduct the surveys. Survey planning relied heavily on village headmen, who notified and gathered the participants on the specified days and times. Results were communicated to the communities and authorities after laboratory analysis of samples was completed.

In villages chosen for MDA, the CE team arrived a few days prior to the beginning of MDA in order to organize and set up new meetings with leaders and villagers to explain the medication, potential side effects, and the regimen that would be followed ^9^. Large, village-scale meetings were combined with group discussions and activities specific for different population groups (e.g. women, school children, farmers or soldiers). During the MDA, the CE team members participated in the MDA process and participated in everyday village life, addressing individual and collective concerns in formal or informal discussions. No specific incentives were provided in exchange for participation, but a mobile clinic was available for all community members during the MDA period, and long lasting insecticide-treated nets (LLINs) were available to those who didn’t already possess them. The MP system allowed for feedback to flow back to headquarters after this seven day period.

In situations where villagers presented concerns or resistance to either surveys or MDA there were further discussions with important community, village and township leaders. A few villagers voiced concerns with regard to loss of blood that were alleviated when medical experts explained that the amount of blood being taken was minimal and would not have an effect on villagers. There were also political concerns since the team signed a memorandum of understanding with the central government and since many of these communities have been involved in a longstanding civil conflict with the military. Through meetings between the CE team and villagers, the villagers came to understand that this program was not from a central government organization, but rather an outside organization that works under the auspices of all locally and nationally relevant organizations. While participation varied in some communities, ultimately no communities completely refused to participate in MDA. Some surveys (15) could not be conducted because of a lack of willing participants. In this case, clinical incidence and consultation rates were closely monitored to ensure that population continued to trust and consult the MP in case of fever.

### Malaria posts (MPs)

**Figure 3.**
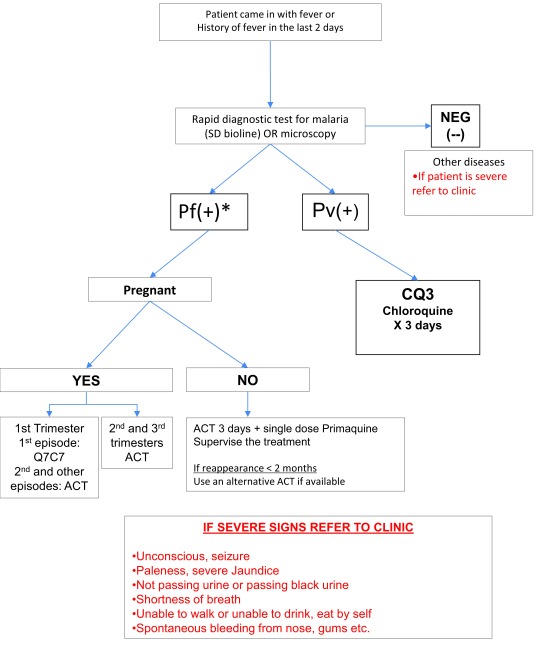
Fever case management algorithm at Malaria Posts. *Pf(+) represents all *Plasmodium falciparum* positive infections, including mixed *P. falciparum* and *P. vivax*. ACT, artemisinin combination therapy.

MPs were established in all communities that accepted the responsibility. An original assumption of 800 villages requiring a malaria post was revised to 1200 following reconnaissance efforts. Physical structures were not provided, but each MP had a trained, dedicated and salaried MPW, antimalarials, rapid diagnostic test (RDT) kits, and a few other basic supplies, such as paracetamol, pregnancy tests, scales, a banner to signal MP location, and stationery. MPWs were selected by the village headman and the community, and attended a five-day training that covered malaria case management, referral and reporting systems and CE. Trainings were followed by a course completion test. A manual (in Karen and Burmese) was provided for reference, summarizing procedures, treatment algorithms ( Figure 3) and dosing tables ( Supplementary File 1).

Fever cases were systematically tested using a *P. falciparum*- *P. vivax* RDT (SD Bioline P.f/P.v, Standard diagnostics/Alere, Republic of Korea). Uncomplicated *P. falciparum* infections were treated with a fixed dose formulation of artemether - lumefantrine (AL) for three days (5 to 24mg/kg of artemether and 29-144mg/kg of lumefantrine). Pregnant women were treated with quinine clindamycin (quinine 10mg/kg and clindamycin 5mg/kg TID) for seven days in the first trimester and AL in the second and third trimesters of pregnancy. A single low dose of primaquine (0.25 mg/kg) was given to prevent further transmission, except in cases of pregnancy, children younger than six months and lactating mothers. Chloroquine 25mg base/kg over three days was used for the treatment of *P. vivax*. Administered doses were determined by the patient’s body weight. An additional blood sample was collected on filter-paper (3×1cm diameter dried blood spots) from *P. falciparum* positive cases in order to monitor antimalarial drug resistance profile.

MP data were sent on a weekly basis, with each MP reporting: all cases of fever by age groups (0-4y, 5-14y and ≥ 15y); all RDT results by age groups (0 – 4, 5 – 14, 15 plus); all malaria treatments by species, gender and age group; the number of severe malaria cases referred, the number of pregnant women with malaria, and the number of deaths attributable to malaria; remaining stocks of artemisinin combination therapies (ACTs) and RDTs ( Supplementary File 2).

At the beginning of the project there was little-to-no cell phone coverage in the entire target area, but by 2017 most of the target area (excluding parts of Hpapun and Hlaingbwe Townships) was covered. Data collection in areas with no cell phone coverage relied on runners who collected the forms and transported them (using any convenient transportation means) to the nearest place where they could be entered into an online data system ( Voozanoo). In areas where access to a GSM (Global System for Mobile Communications) network was available, MP weekly data reports were entered using a smartphone application specifically developed using Android open source materials (DroidDB and BarcodeScanner, Syware Inc, free runtime developer license 4.1). Used on relatively inexpensive smartphones (80 US$), this application allowed to scan the MP identification code from a barcode sticker thus limiting typing errors, to capture the data in an entry interface matching the reporting form, and to convert it in a standardized format SMS (short messaging service, e.g. text message). In the receiving phone, SMS were extracted as an Excel file (SMS To File Free_1.2_3) and aggregated to the database. After aggregation, MP data completion and correctness was regularly assessed by searching for duplicate reports, missing weekly reports, through double entry of a subset of records and through integrated weekly GIS routines that link records to spatial references.

MP activities were continuously monitored via the weekly data reporting system and by a dedicated monitoring and evaluation team, which was recruited to make investigative visits, using standardized questionnaires ( Supplementary File 3), to randomly selected MPs and MPs that ceased to transmit data, or reported no activity or stock outs. Every month, systematic quality control of RDTs from 15 randomly selected MPs was performed at central headquarters in Mae Sod, Thailand ( Figure 1). A data evaluation algorithm was established in order to monitor data quality. Missing reports, abnormal data reporting, spikes in clinical malaria cases, or potential problems in stock inventories were checked weekly. After potential data entry errors were excluded, the malaria post supervisors in charge of any problematic MPs were contacted to assess the situation and the required response.

### Malaria prevalence surveys

***Procedures.*** Cross sectional surveys were conducted at the village level using an ultrasensitive high-volume qPCR assay in order to estimate the prevalence of *P. falciparum* and *P. vivax* malaria ^8^. To ensure that the selected villages were representative of the area, a grid (with 20km wide by 30km long cells) was superimposed on a map of the target area and each cell of the grid was assigned a number of surveys, sufficient to reach roughly 25% survey coverage (from an original village count of 1000). Villages within each grid cell were then randomly selected using sampling functions in STATA v14.1 (“sample”) and R v3.4.0 (“sample()”). Surveys were originally planned in 250 randomly selected villages. An additional 60 suspected hotspot villages were included later, based on geographic proximity to a detected hotspot and/or higher-than-expected incidence given the duration of MP activity in the village, leading to oversampling in high prevalence areas. Follow-up cross sectional surveys were conducted > 12 months post-MDA to assess the impact following the same procedures.

Following CE, survey teams approached village headmen to aid in selecting villagers for possible participation in a survey. In most cases no village census was available. Survey teams took samples from adults who agreed to participate, attempting to balance samples across sex and broad age groups, until reaching the sample size needed based on the full village population ( Supplementary File 4 and Supplementary File 5). This sample size represented a significant proportion of the village population. Assuming that 50% of inhabitants were older than 18, which was verified in complete census obtained during MDA interventions, the sample size often comprised between 30 and 50% (baseline) and 50–80% (M12) of adult village population ( Supplementary File 5). In follow up surveys (> M12) and in very small villages (e.g. 20 houses) it was necessary to sample multiple people from the same household. Participants were asked to provide 2mL blood by venous puncture after providing written informed consent. Samples were collected in EDTA (Ethylenediaminetetraacetic acid) tubes, stored in an icebox and brought back to the field laboratory within 48h of collection. A RDT was performed in the field for all participants to the survey, and proposed to the rest of the village population. All RDT-positive malaria cases were provided with adequate treatment by the MP. An additional blood sample was collected on filter-paper (3×1cm diameter dried blood spots) from *P. falciparum* RDT-positive cases in order to monitor antimalarial drug resistance profile.

***Malaria detection in the laboratory.*** Upon arrival in the laboratory an aliquot of whole blood was used for malaria detection by RDT (SD Bioline P.f/P.v) and microscopy (thin and thick smears). The remaining blood was centrifuged and packed red blood cells were used for extraction and detection of malaria parasites by ultrasensitive qPCR ^8^. Briefly, 500 μl packed red blood cells were manually extracted using QIAamp DNA blood midi kits (Qiagen) and rehydrated in 25 μl of the provided AE buffer. *Plasmodium* specific monoplex PCR assays were performed using an ABI 7500 fast machine (Applied Biosystems) with five-fold serial dilutions of *P. falciparum* 3D7 standard at dilutions of 50,000 to 16 parasites/ml. The serum, buffy coat and residual packed red blood cells were kept at -80°C.

The detection by qPCR was conducted in two steps: 1) detection of Plasmodium infection using a highly sensitive genus-specific marker; 2) determination of *P. vivax* or *P. falciparum* species in Plasmodium positive samples using less sensitive species-specific markers ^17^. The species of some Plasmodium positive samples could therefore remain uncharacterized. In these instances microscopy and RDT results were used to attribute the species. If both RDT and microscopy were negative, malaria was attributed to either *P. falciparum* or *P. vivax* according to the relative proportion of each species already detected in samples with complete qPCR results.

***Sample and data management.*** Individual samples were identified using a barcode label that was used to trace field data and results obtained for the different malaria detection tests performed in the laboratory (RDT, microscopy, qPCR). All paper-recorded survey data (participant demographic information, field RDT result, laboratory RDT and microscopy examination results) were entered in a Microsoft Access (Access 2010 version 14.0) database. Result outputs from qPCR analysis were merged directly in the Access database. Double entry of survey results was performed for 39/272 baseline surveys (14%) and ten percent of the remaining results were checked to confirm minimal error rates.

***Sample size.*** The within-village sample size was calculated, taking into account feasibility constraints (small village sizes, sample conservation and cold chain) and the expected precision of estimates. For baseline surveys (month zero, M0), the targeted sample size was calculated to measure a 40% malaria prevalence with a +/-10% precision of the 90% CI ( Supplementary File 4). For M12 (month 12) surveys, the targeted sample size was calculated to measure a 90%-decrease from baseline *P. falciparum* prevalence ( Supplementary File 5). If the expected M12 prevalence was ≤2%, the sample size was calculated to achieve a 95% CI-width of +/-100% of expected value. If the expected M12 prevalence was >2%, the sample size was calculated to achieve a 95% CI-width of +/-50% of expected value (e.g. for a village with baseline prevalence 30%, M12 survey aimed at a sample size sufficient to measure a 3% prevalence with a 95% CI interval of +/-1.5%).

***Hotspot definition.*** A village was operationally classified as a malaria “hotspot” when the 90% CI upper limit of the sum of *P. falciparum* and *P. vivax* prevalence estimate was ≥40% and the corresponding value of the proportion of *P. falciparum* in the positive samples was ≥20%. Such villages were targeted for mass drug administration.

### Targeted mass drug administration (MDA)

***Drug regimen and exclusion criteria.*** The ACT regimen used in MDA consisted of dihydroartemisinin (7mg/kg) plus piperaquine (55 mg/kg) (DP) with a single low dose of primaquine (0.25mg/kg). Women in their first trimester of pregnancy, children under one year of age, individuals with previous drug allergies and villagers who refused to participate were excluded from MDA. Women within reproductive age (roughly 14 – 44 years old) and of unknown pregnancy status (self-reported being unsure of pregnancy status) were screened with a urinary human chorionic gonadotropin (HCG) test kit. Women in their second and third trimester of a pregnancy, as well as breastfeeding mothers, were eligible for DP treatment but were excluded from the single dose of primaquine.

***Procedures.*** After obtaining written informed consent, each eligible participant’s medical history was briefly reviewed and a clinical examination was conducted. Those who met the inclusion criteria were administered a three-day course of DP with a single low dose of primaquine on the first day and this was repeated over three consecutive months ( Supplementary File 3). All doses were directly observed. Participants who were unable to attend to the next take(s) were provided the remaining doses to complete unsupervised treatment. The MDA team stayed in each MDA village for seven days per visit to document side effects, to address concerns and to treat other minor illnesses. All adverse events (AE) that were reported by MDA participants within one week of taking an MDA course were carefully recorded and treated when necessary at a mobile clinic set up during the MDA period or referred to the nearest appropriate health care facility. All drug administration data (date, weight, dosage) were recorded in a logbook as well as reasons for non-participation collected using a standardized questionnaire ( Supplementary File 6). All MDA data were checked onsite by the field teams and reviewed after the end of the 3 months. Logbooks and AE sheets were entered in an Access database (Access 2010 version 14.0).

MDA participation was calculated as the total number of individuals completing one, two or three 3-day treatment courses over the 3 months of MDA intervention, divided by the total number of individuals recorded as present in the village at least on one occasion during the three months of activity, excluding visitors staying <2 weeks. MDA efficacy was assessed by prevalence surveys conducted ≥ 12 months after the start of MDA and by monitoring the incidence of clinical episodes at the MP.

### Antimalarial resistance monitoring

Antimalarial resistance monitoring was conducted at two partner laboratories: one in the Faculty of Tropical Medicine at Mahidol University and the other at the Sanger Institute, both using dried blood spots from *P. falciparum* positive cases at MPs.

***Assessment of mutations in PfKelch13 (associated with artemisinin resistance).*** Polymorphisms in the *PfKelch* gene were assessed by nested PCR amplification covering the full length of the gene (total 2181 bp) ^18^, followed by DNA sequencing using an ABI sequencing platform (Macrogen Inc, South Korea). Cross contamination was monitored by adding negative control samples in every run. Sequencing results were aligned against *PfKelch13* of reference strain 3D7 (putative 9PF13_0238 NCBI Reference Sequence (3D7): XM_001350122.1), using Bioedit software (Abbott, CA, USA). Polymorphic patterns were assessed by two individuals blinded to the origin of the sample.

***Markers of ACT partner drug resistance.*** *PfPlasmepsin2* and *Pfmdr1* copy numbers were quantified using Taqman real time PCR on a Corbett Rotor-Geneä Q (Corbett Research, Australia), following previous reports ^18, 19^. Amplification was performed in triplicate on a total volume of 10 µL as multiplex PCR using Quantitec Multiplex PCR no ROX (QIAgen, Germany). Every amplification run contained 9 replicates of calibrators and triplicates without template as negative controls. β-tubulin served as an internal standard for the amount of sample DNA added to the reactions. Copy numbers were calculated using the formula: copy number= **2 ^-DDCt^** ; with **DD C _t_** denoting the difference between D C _t_ of the unknown sample and D C _t_ of the reference sample.

Tests for piperaquine resistance markers were also carried out on *P. falciparum* DNA sequence data from 216 clinical samples collected in the same region between 2013 and 2015, included in the MalariaGEN *P. falciparum* Community Project. DNA was extracted directly from blood samples taken from patients at admission time, after leukocyte depletion by CF11 filtration to minimize human DNA. Selected samples, having >50 ng DNA and <80% human DNA contamination, were sequenced on the Illumina HiSeq platform following the manufacturer's standard protocols ^19^. Paired-end sequencing reads of length 200–300 bp were obtained, generating approximately 1 Gbp of read data per sample. Polymorphism discovery, quality control and sample genotyping followed a process described in detail elsewhere ^20^. Three tests for piperaquine resistance markers were performed, and samples were considered sensitive if all three tests yielded negative results: 1) Position 2,504,560 on chromosome 10 was genotyped to assess the presence of the exo-E415G mutation ^21^; 2) Sequencing reads were searched for the breakpoint sequence (ATGATTACGATAATCACACTGTTGGTTTCGCCCTT) that characterizes plasmepsin 2-3 amplifications associated with piperaquine resistance in Cambodia ^21^; and 3) Copy number was assigned to plasmepsin 2-3 from a genome-wide analysis of sequencing read coverage, using a procedure based on a Gaussian hidden Markov model (HMM), described in detail elsewhere ^22, 23^.

The same sequencing data were used to estimate copy number for the *pfmdr1* gene, using a method previously described in detail ^24^. Briefly, the sequencing read coverage was normalized for each sample, and the *pfmdr1* copy number was estimated by calculating the ratio between the coverage at a number of positions within that gene, and the median coverage of a set of 56 reference positions at various loci across the genome. To improve estimates, the reference positions were chosen in genes with similar characteristics to *pfmdr1*: similar GC content, level of evolutionary conservation, exon length, median coverage, and low variation in relative coverage across the MalariaGEN dataset. Each sample’s reference coverage was estimated as the median of coverage at the 56 positions, while seven positions in *pfmdr1* were analogously used to determine *pfmdr1* coverage, and hence the copy number estimation.

### Statistical analyses

Incidence rates of clinical *P. falciparum* or *P. vivax* malaria episodes (cases per 1,000 population per unit of time) and 95% Poisson confidence intervals were calculated using weekly MP data reports. Weekly incidence was calculated for each village and also aggregated over space and time (e.g. by month or year and by village tract or by township).

Total malaria, *P. falciparum*, and *P. vivax* prevalence and 95% binomial Wilson confidence intervals (corrected for finite population size) were calculated using the results of surveys analyzed by high volume ultrasensitive qPCR.

Statistical clustering of both incidence and prevalence was assessed using a range of geostatistical and cartographic approaches. Analytic approaches for analyzing spatial autocorrelation of population standardized incidence and prevalence data included the global Moran’s I statistic, local indicators of spatial autocorrelation, Kulldorff’s scan statistic ^25^, variography and spatial correlograms. It was necessary to carefully interpret results and to look for congruity between approaches because of the skewed nature of the data (with many villages having low prevalence and few having high prevalence). Choropleth maps were created for incidence at the village tract level and point maps were created and used for operational needs (e.g. to design stocking or survey strategies and to assess potential gaps in health care coverage). Statistical analyses were done using STATA v14.1 (STATA Corp), R v3.4.0 (The R Foundation) and the Spatial Analysis in Macroecology software package (v4.0).

The evaluation of the impact of the program was conducted by monitoring the temporal trends in incidence at township, village tract and village level and by measuring the proportion of villages and village tracts achieving and sustaining low *P. falciparum* case incidence. The specific impact of MDA on malaria prevalence in hotspot villages was measured by comparing the baseline prevalences to follow-up survey prevalences 12 months after MDA.

## Discussion

This project illustrates the scale up of an elimination program in a region encompassing remote and rugged terrain, a complex political landscape, ongoing areas of active conflict, and a near-absent pre-existing health care infrastructure. The key interventions of the project included the establishment of a dense network of community level early malaria diagnosis and treatment clinics (MPs) and targeted MDA.

There are several limitations to this protocol. This program is organized at the village level, which is an operationally relevant unit for programming and implementing interventions, but may overlook malaria dynamics at higher scales (e.g. hotspots encompassing more than a single village). This issue was addressed in part by surveying neighbouring villages of hotspots in order to define their actual size. The definition of hotspots was based on assumptions from previous surveys and studies in the region. It is probably useful to revisit and refine it in the light of the results obtained at large scale by this program. Geostatistical approaches such as the use of Kulldorff’s scan statistic ^25^ may be useful for defining hotspots of infections and drug resistant strains.

Other limitations were related to operating a large program in remote and politically complicated areas. Monitoring the impact of the project relied mostly on observational data from the MPs, with the exception of specific MDA activity, for which additional surveys were conducted. More detailed studies were not possible because of the magnitude of the intervention area, as well as occasional barriers to accessing field sites because of active conflict, natural disasters or political sensitivities.

Another major operational constraint was that interventions (MP openings; baseline surveys; MDA) could not be simultaneously conducted in all locations across the target area. As a result, for example, baseline surveys could not be conducted at the same season or after the same duration of MP opening in all villages. Likewise, MDA was performed before one of the two main transmission seasons (rainy season or cold season), but not necessarily the same season. There was no specific follow-up of control villages, since the operational goal was elimination and given potential ethical implications of not treating hotspot communities. The step-wedge nature of the deployment will, however, allow some degree of comparison of incidence between villages with MDA occurring sooner or later after MP opening.

The prevalence surveys, which relied on an ultrasensitive qPCR approach, posed a significant challenge or constraint for this program. Blood samples needed to be quickly processed at a laboratory meaning that particularly remote areas with difficult access were difficult or impossible to survey. The ongoing development of a new generation of high sensitivity RDTs could dramatically simplify the measurement of *P. falciparum* prevalence ^26^. Likewise, incidence-based or risk-based targeting of prevalence surveys could also narrow down surveys to suspected hotspots. In a programmatic setting, these would allow for more efficient, faster, and decentralized decision-making with regard to interventions other than MPs.

The project relied on an adaptive strategy, focussing on the rapid establishment of a geographically referenced health care infrastructure (the MP network), drawing from on-the-ground knowledge of the area (CE and geography), utilising existing systems when possible (community-based partners) and quickly filling gaps where necessary. The project has now entered its fourth year and results from the first three years will be published soon. It was important to find a balance between approaches that were scalable but also took into account the local contexts and complexities of the target area. The strategies employed here and lessons learned through this project can be applied to eliminate malaria in other settings and for other infectious disease elimination programs.

## Ethical statement

All individuals participating in blood surveys and MDA provided written informed consent. Consent forms were translated into S’kaw Karen, the most commonly spoken native language in the target area. The forms were back-translated by native speakers at SMRU and corrected when necessary prior to use in the field. All CE team members are fluent in the language and were able to explain the forms and the project in S’kaw Karen to villagers.

This project was approved through the Ethics Review Committee on Medical Research Involving Human Subjects from the Republic of the Union of Myanmar, Ministry of Health and Sports, Department of Medical Research (Lower Myanmar): 73/Ethics 2014.

## Supplementary material

Supplementary File 1: Malaria post manual (in English).

Supplementary File 2: Weekly Malaria Post data report form.

Supplementary File 3: Malaria post monitoring and evaluation questionnaire.

Supplementary File 4: Table for baseline qPCR survey sample size calculation.

Supplementary File 5: Table for M12 qPCR survey sample size calculation.

.

Supplementary File 6. Mass Drug Administration data collection logbook (reduced from A3 format) is shown in *part A*. Having all 3 months of follow-up on the same sheet was important for the teams to be able to follow participation accurately. This included drug uptake and gathering standardized data on non-participation, detailed in *part B*.
