# Supplementary material for: Scale up of a Plasmodium falciparum elimination program and surveillance system in Kayin State, Myanmar
Source: Wellcome Open Res. 2017 Dec 22;2:98. Originally published 2017 Oct 9. [Version 2] doi: 10.12688/wellcomeopenres.12741.2 (PMC5701446; doi:10.12688/wellcomeopenres.12741.2)
Supplement: Supplementary file 1 [file wellcomeopenres-2-14723-s0000.tgz › 4a33d2ed-8bd6-4bfc-b395-d1500ec79461.pdf]

MALARIA ELIMINATION TASK FORCE

MALARIA POST WORKER

HANDBOOK

VILLAGE NAME:

____

VILLAGE CODE: (for RDTs and forms)

___ ___ - ___ ___ ___

WORKERS NAME:

____

Manual V 3.1 (Eng)(Feb 2016)

1. Acronyms	3
2. METF introduction & Community engagement	4
3. Objective and function of malaria post	6
4. Background of malaria<br>- malaria species, life cycle, transmission, prevention	7
5. Malaria symptoms and complication	8
6. Differential diagnosis of malaria	9
7. Instructions for malaria RDT procedure	10
8. Malaria treatment	11
9. Precautions	19
10. Records and reporting sheets	
11. Inventory lists for Malaria post.	

Acronyms

RDT – Rapid diagnostic test (SD bioline)
ACT – Artemisinin based combination therapy
COA3 - Coartem
DP – Dihydroartemisinin-piperaquinine
MAS3 - Artemether-lumfantrine
Q7 C7 – Quinine and Clindamycin
PQ – Primaquine
CQ - Chloroquine
Doxy - Doxycycline
MP – Malaria post
P.F – plasmodium falciparum
P.V – plasmodium vivax
G6PDd – Glucose 6 phosphate dehydrogenase deficiency
POS - positive
NEG - negative
od – Once daily
bd – Two times a day
tds – three times a day
qid – four time a day
mg – milligram
kg – kilogram
ml – milliliter
D1 – Day 1
D2 – Day 2
D3 – Day 3

**1 tablet**	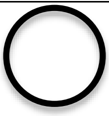
**¼ = one quarter of tablet**	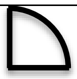
**½ = half tablet**	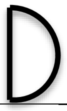
**¾ = three quarters of a tablet**	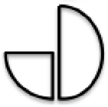

Malaria Elimination Task Force (METF)

The Malaria Elimination Task Force (METF) aims to develop and implement methods to eliminate malaria rapidly in Eastern Myanmar. The initial target is the elimination of artemisinin resistant falciparum malaria. The METF started activities in May 2014 in four townships in eastern Myanmar (Karen/Kayin state).

The strategy of the METF rests on seven pillars:

1) *Mapping and geographic information system*
   Knowing the terrain is essential so detailed and reliable mapping of villages is the first priority. Maps are used for the planning and monitoring of all subsequent activities.

2) *Community engagement*
   This malaria elimination programme requires a minimum understanding of the disease as well as support of the population for the proposed activities. Trust is at the heart of the Community Engagement (CE)

3) *Malaria posts*
   The functioning and effective malaria post (MP) is key to *P. falciparum* elimination: the MP is a simple structure that provides free and uninterrupted access to reliable diagnosis and effective treatment for any clinical malaria cases from the community within 24-48 hours of fever onset.

4) *Real-time data collection and reporting*
   To achieve prompt elimination of *P. falciparum* malaria, intelligence in real-time is crucial to adapt and respond effectively to a rapidly changing front-line. Weekly data are needed to assess MP activities, responsiveness, supplies and impact.

5) *Submicroscopic malaria prevalence surveys*
   There is now robust evidence that a substantial proportion of the population in endemic areas harbour malaria parasites without apparent illness. Identifying and treating these reservoirs is essential for the rapid elimination of *P. falciparum*.

6) *Mass drug administration*
   MDA has two objectives: to eliminate the reservoir of parasites rapidly and to provide protection against new infections for at least a month after each round of MDA.

7) *Entomology*
   A good understanding of the vector population and its behaviour is important for malaria elimination. Detailed entomological studies were conducted prior to the start of the METF programme.

Objective of functioning malaria post

1. To detect and treat malaria in 24 hours
2. To control malaria spreading/transmission
3. To stop drug resistant malaria spreading to the whole region
4. To be there for people who suffer from malaria

Job Description of Malaria Post Worker (MPW)

Malaria post worker trained to;

- Conduct and interpret rapid test results
- Observe universal precautions
- Record result
- Report result
- Knowledge on where to refer for severe cases
- 2 person got alternative duty/present

Task: of MPW (malaria post worker)

- Stay in village and treat malaria within 24 hours
- Administer drugs correctly to patients
- Recognize side effect and patient condition
- Referral severe case to nearest health center
- Good communication with local NGO in collaboration
- Checking stock and conduct stock inventory on regular basic
- Make sure supplies in stock are up to date and enough for at all time
- Keep working environment clean and documents safely (all document consider confidentiality)
- Detect and report all malaria cases and outbreak situation to supervisor

Functioning Malaria post

- Clean place, MPWs household or other
- Storage room or shelves with proper lock
- Continuous supply of RDTs, ACTs, data forms and MP worker
- Correct diagnosis of malaria
- Correct treatment of malaria
- Timely data return.

Community engagement

Disease elimination is not possible without the participation of the population. This malaria elimination programme requires a minimum understanding of the disease as well as support of the population for the proposed activities. Trust is at the heart of the Community Engagement (CE), a complex and difficult process. METF has benefited from the help of social scientists but the activities are carried-out mainly by people from the affected communities who organize meetings first with the leaders then with the entire population. Aids such as handbooks, leaflets and posters have also been developed.

No.	Information to convey	Targeted population	Method
1	Notify to the respective authoritative person about the training completion of MP	Village Head Man	Notifying
2	Inform the villagers about opening of malaria post in the village	Villagers	Advertising
3	Educated the patients to test for malaria within 24 hours of fever	Villagers	Counselling

What is malaria?

Malaria is caused by infection of red blood cells with protozoan parasites of the genus *Plasmodium* inoculated into the human host by a feeding female anopheline mosquito.

Plasmodium species

1. Plasmodium falciparum
2. Plasmodium vivax
3. Plasmodium ovale
4. Plasmodium malariae
5. Plasmodium knowlesi

Life cycle and transmission

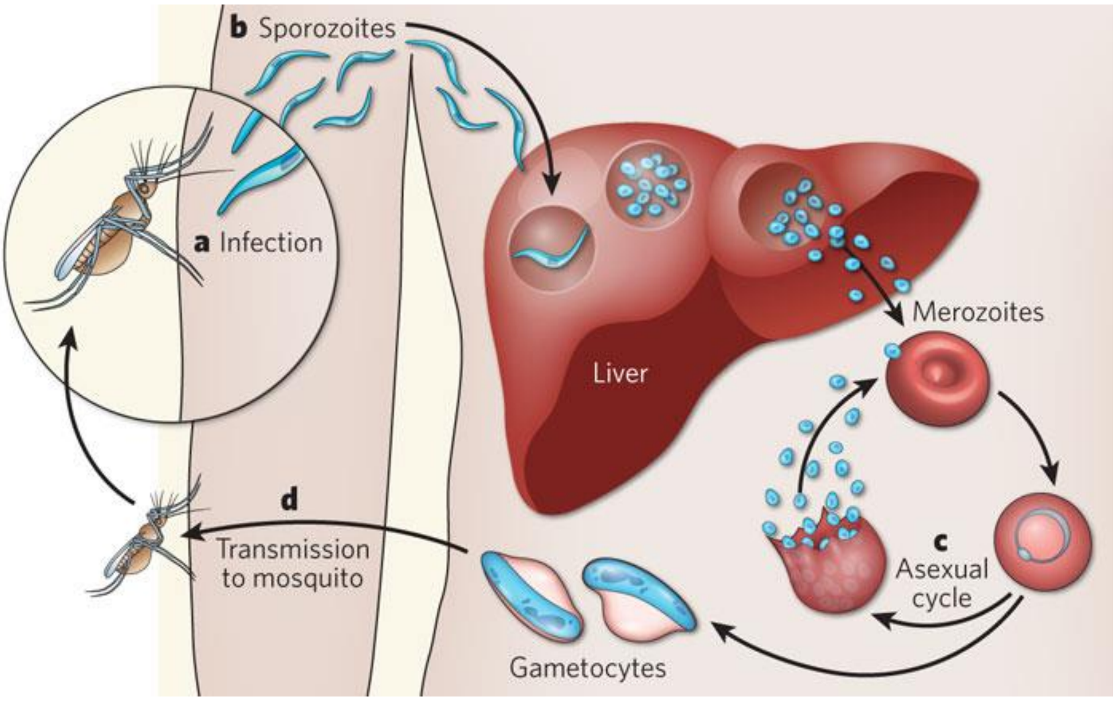

In humans, after being bitten by an infected mosquito the malaria parasites first infects the liver then the red blood cells are infected next, at this stage symptoms of malaria appear.

When an uninfected mosquito bites an infected human they become infected and continue the cycle.

<u>Transmission</u>

Malaria is transmitted by the bite of infected female Anopheles mosquitos.

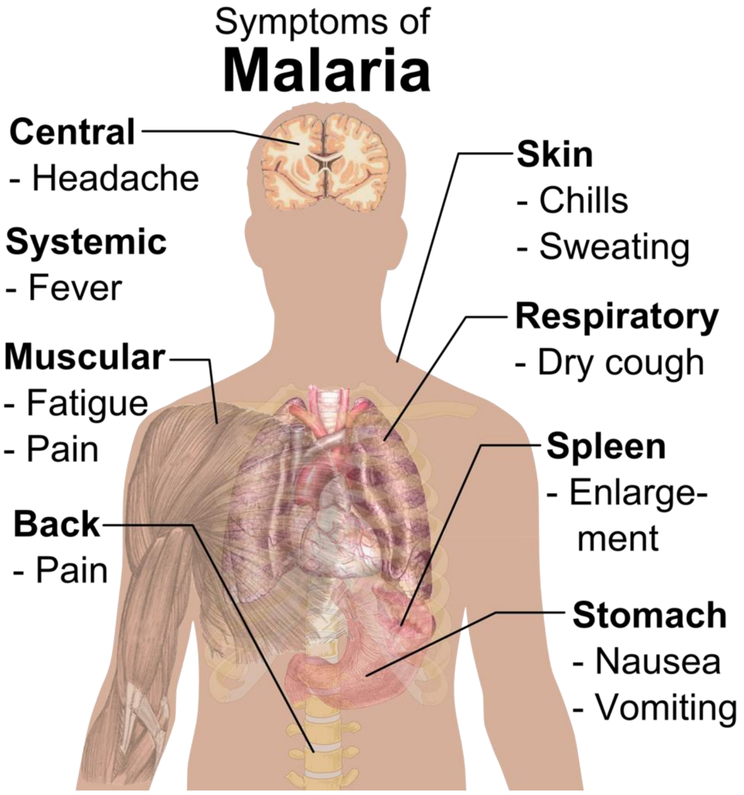

Symptoms of uncomplicated malaria

1. Fever
2. Chills and sweating
3. Headache
4. Muscle pain
5. Nausea, vomiting
6. Fatigue

Severe malaria symptoms

1. Unconsciousness, fits, coma
2. Very pale, yellow coloration of the skin and eyes
3. No passing of urine in 24 hours or passing of dark urine
4. Difficulty in breathing
5. Unable to walk or drink or eat by oneself
6. Bleeding from nose, gum, etc

Complication

If left untreated, severe malaria is fatal in the majority of cases. (Cerebral malaria, renal failure, pulmonary edema, hypoglycemia, severe anemia)

Prevention

- Cover exposed skin by wearing long-sleeved shirts, long pants and hats especially when you go into the forestry area at the evening and night time.
- Use an appropriate insect repellent.
- If possible, sleep under the insecticides treated nets
- Test for the malaria at clinics if fever developed.

Differential diagnosis of malaria

Dengue

High fever, possibly as high as 105°F (40°C), pain behind the eyes and in the joints, muscles and/or bones, severe headache, rash over most of the body, mild bleeding from the nose or gums, bruising easily.

Flu (Common cold)

Fever* or feeling feverish/chills, cough, sore throat, runny or stuffy nose, muscle or body aches, headaches, fatigue (tiredness).

Enteric fever (Typhoid)

Fever often up to 104 F, poor appetite, abdominal pain, headaches, generalized aches and pains, lethargy (usually only if untreated), intestinal bleeding diarrhea or constipation.

Hepatitis

Yellowing of your skin and eyes (jaundice), abdominal pain, dark urine, fever, joint pain, loss of appetite, nausea and vomiting, weakness and fatigue.

Meningitis

Severe headache, fever, stiff neck confusion and irritability, vomiting, drowsiness and difficulty waking up, severe muscle pain, pale, blotchy skin, and a distinctive rash (although not everyone will have this).

UTI

A burning feeling when urinate, a frequent or intense urge to urinate, pain or pressure in your back or lower abdomen cloudy, dark, bloody, or strange-smelling urine, feeling tired or shaky, fever /with chills.

Others – Pneumonia, Scrub typhus, Leptospirosis, etc.

Interpretation of Malaria RDT test results

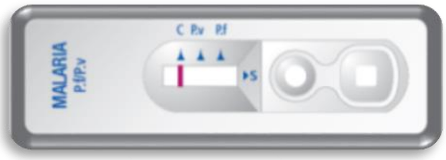	**NEGATIVE -** ပိုးမရှိခြင်း၊
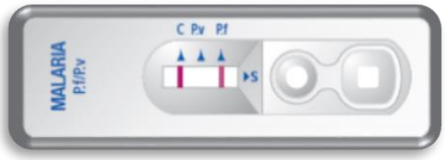	**POSITIVE – Falciparum malaria (P.f)**<br><br>ပြင်းထန်ငှက်ဖျားပိုးရှိခြင်း၊
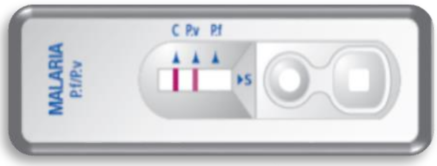	**POSITIVE – Vivax malaria (P.v)** ပိုးရှိခြင်း၊
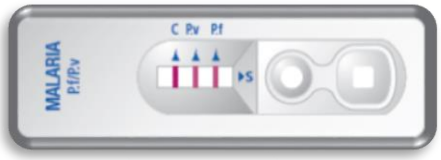	**POSITIVE – Mixed infection (P.f,P.v)**<br><br>ပိုး နှစ်မျိုးလုံးရှိခြင်း၊
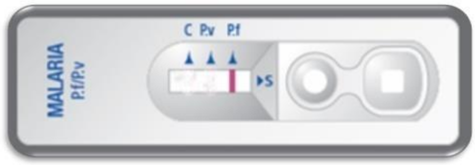<br><br>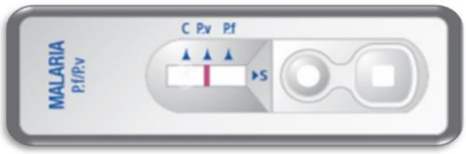	**Control -** မျဉ်းကြောင်းလိုင်းများ မပေါ်ခြင်း<br><br>**Invalid – repeat the test** နောက်ထပ်တစ်ခါ<br><br>အသစ်ပြန်ဖေါက်ရမည်။ (RDT) အသစ်သုံးရမည်။<br><br>**Invalid – repeat the test**

Management

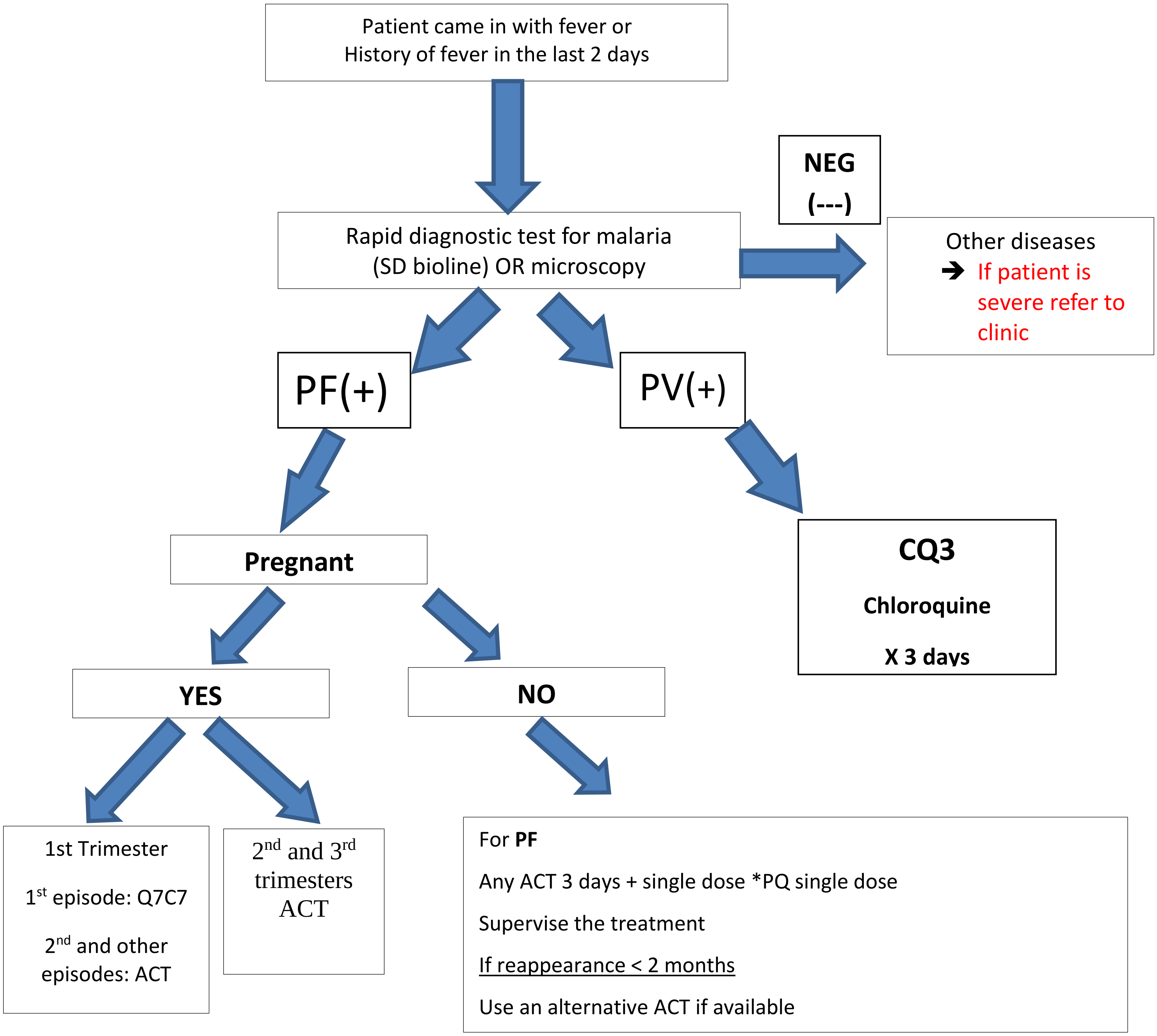

IF SEVERE SIGNS REFER TO CLINIC

- Unconscious, fitting
- Very pale, Severe Jaundice
- Not passing the urine or black urine
- Shortness of breath
- Unable to walk or unable to drink, eat by self
- Spontaneous bleeding from nose, gum etc.

If PF+ patient ≥ 44 kg

Artequin; MAS3. Give dose once daily for 3 days

	Mefloquine 3	Artesunate
Weight	8 mg/kg(OD)	4mg/kg (OD)
(kg)	tab	tab
44-46	1- 1/2	1
47-48	1- 1/2	
49-51	1- 3/4	
52-53	1- 3/4	
54-56	2	
57-58	2	1 1/4
59-61	2	
62-63	2	
64-66	2- 1/4	
67-68	2- 1/4	
69-71	2- 1/4	1 1/2
72	2- 1/2	
73-77	2- 1/2	
78	2- 3/4	
79-81	2- 3/4	

DP3

PF+ Give dose once daily for 3 days

Weight(kg)	Tab	ml
5		1.3 ml
6		1.6 ml
7		2 ml
8-12	1/2	
13-20	1	
21-30	1- 1/2	
31-40	2	
41-50	2- 1/2	
51-60	3	
61-70	3- 1/2	
71-84	4	
85-100	5	

A suspension is made by allowing 1 tablet to dissolve in 5ml clean water.

Coartem (ကိုအာတမ္)

COA3; PF+ Give dose twice a day for 3 days

**Weight** (kg)	**tab**
≤15	1 per dose
16-25	2 per dose
26-35	3 per dose
>35	4 per dose

ကိုယ္အေလးခ်ိန္ (kilogram)	ပထမေန႔		ဒုတိယေန႔		တတိယေန႔	
	မနက္	ည	မနက္	ည	မနက္	ည
≤ ၁၅	○	○	○	○	○	○
၁၆ -၂၅	○○	○○	○○	○○	○○	○○
၂၆ - ၃၅	○○○	○○○	○○○	○○○	○○○	○○○
> ၃၅	○○<br>○○	○○<br>○○	○○<br>○○	○○<br>○○	○○<br>○○	○○<br>○○

<u>Need to take with some fried or oily food or a carton of flavored milk.</u>

<u>အစား စားၿပီးမွ ေဆးေသာက္ ပါ။</u>

Q7C7 (ကီြနင္း + ကလင္ဒါမိုင္စင္)

For PF+ Pregnant Women 1st trimester 1st attack
Oral quinine and Clindamycin, both three times a day for 7 days

Weight (Kg)	Quinine	Clindamycin
15-18	½	1
19-26	¾	1
27-33	1	1
34	1- ¼	1
35-41	1- ¼	2
42-48	1- ½	2
49-56	1- ¾	2
57-63	2	2
64-69	2- ¼	2
70-71	2- ¼	3
72-78	2- ½	3
79-86	2- ¾	3

If develops severe diarrhea stop clindamycin

Primaquine (7.5 mg tablet)

PQ (ပမ်ုကြင်း)

For all PF+ patients; 1 dose, on day 1 of ACT EXCEPT pregnant women, breastfeeding women and children < 6 months

Dose: **Single dose**

Wt	Tab	ml
4		0.7mL
5		0.8mL
6		1.0mL
7		1.2mL
8		1.4mL
9		1.5mL
10		1.7mL
11-19	1/2	
20-26	3/4	
27-33	1	
34-41	1- 1/4	
42-48	1- 1/2	
49-56	1- 3/4	
57-63	2	
64-70	2- 1/4	
71-78	2- 1/2	
79-84	2- 3/4	
85-	3	

A suspension is made by allowing 1 tablet to dissolve in 5ml clean water (1ml=1.5mg)

Use tablet cutter to cut tablets.

Give food before dose to prevent abdominal pain and nausea

For PV patients (P.v လူနာ)

Chloroquine (ကလိုရိုကြွင်း)

CQ3; Give dose once daily for 3 days.

Weight (kg)	D1 &D2	D3
3-5	1/4	1/4
6-9	1/2	1/4
10-11	3/4	1/4
12	3/4	1/2
13-17	1	1/2
18-19	1- 1/4	1/2
20	1- 1/4	3/4
21-25	1- 1/2	3/4
26-27	1- 3/4	3/4
28	1- 3/4	1
29-33	2	1
34-35	2- 1/4	1
36	2- 1/4	1 1/4
37-41	2- 1/2	1 1/4
42	2- 3/4	1 1/4
43-44	2- 3/4	1 1/2
45-48	3	1 1/2
49-50	3- 1/4	1 1/2
51-52	3- 1/4	1 3/4
53-56	3- 1/2	1 3/4
57	3- 3/4	1 3/4
58-60	3- 3/4	2
61-64	4	2
65-66	4- 1/4	2
67	4- 1/4	2 1/4
68-72	4- 1/2	2 1/4
73	4- 3/4	2 1/4
74-75	4- 3/4	2 1/2
76-79	5	2 1/2
80-82	5- 1/4	2 1/2

Ferrous sulphate and folic acid dosing (for anaemic patient)

14 days treatment.

**Age group**	**Ferrous sulphate**<br>**Tab**	**Folic acid**<br>**Tab**
>12 year	1 three times a day	1 once a day
5-12 Year	1 ½ once a day	1 once a day
1-4 year	1 once a day	1 once a day
<1 year	½ once a day	½ once a day

Special considerations

Treatment

- Always use the weight-base regimen for antimalarial treatment. (Use the dosing tables provided)
- If Coartem is to be used, give together with fatty food, oil or milk.
- Stat dose of primaquine 0.25 mg/kg is given on the first day of every PF treatment for prevention of further transmission. Give food with primaquine.

For woman,

- Ask if the woman is pregnant. If unsure conduct pregnancy test if available.
- 1st attack use Q7C7 for first trimester or unsure gestation.
- All other pregnant women use AS 7C7 for second or third trimester or other ACT.
- DO NOT GIVE Doxy or Primaquine if pregnant or breastfeeding.

Allergy

- Always ask if patients are allergic to medicines (bad reaction in the past?) if yes use a different ACT

- If any allergy occurs during the administration of ACT, ask to stop taking the drug and refer to nearby health center or use another malaria regimen.

Contraindications

- Mefloquine: Previous MAS3 < 2 months, history of convulsions, epilepsy, mental illness
- Primaquine: Pregnant women or breastfeeding, or under 6 months, patient with know history of G6PD deficiency.
- Doxycycline: pregnant women and children under 8 years

Side effect

- Clindamycin; Severe diarrhea; stop clindamycin continue with artesunate
- MAS3, COA3, DP: might give nausea and dizziness (usually mild)

Vomiting

- Vomit <30 min (repeat full dose)
- Vomits >30 and <60 min after (give half dose)
- Vomits >60 min after (no dose)
- **Vomits twice: cool down the patient, wait 15 min and try again. If vomiting again, send to clinic.**
