# Supplementary material for: Scale up of a Plasmodium falciparum elimination program and surveillance system in Kayin State, Myanmar
Source: Wellcome Open Res. 2017 Dec 22;2:98. Originally published 2017 Oct 9. [Version 2] doi: 10.12688/wellcomeopenres.12741.2 (PMC5701446; doi:10.12688/wellcomeopenres.12741.2)
Supplement: Supplementary file 2 [file wellcomeopenres-2-14723-s0001.tgz › 2c51dd75-a54f-4282-b517-d05655009f0a.pdf]

MP Village Name:	MP (village) code: |___|___|-|___|___|___|___|___|
|_________________________________|	Report by: |________________________| name

Malaria Elimination Task Force weekly report form (SD BIOLINE)

***From date:|___|___| |___|___| |___|___| to date: :|___|___| |___|___| |___|___|**

Day *Month* *Year*

*Please count from **MONDAY** to **SUNDAY** always:
စးထိဉ်ဖွးနှၤ်လါမုၢ်တနံၤတုၤလါမုၢ်အိဉ်ဘှံးထီဘိ း

Table 1: Total fever cases ပှၤဆါတၢ်ကိၢ်ထိဉ်(Number of cases of **FEVER** တၢ်ကိၢ်ထိဉ်အနိဉ်ဂံၢ်)

	**Fever cases**
**<5 years**	
**5 to 15 years**	
**Older than 15 years (>15)**	
**Total**	

Table 2: Laboratory results တၢ်မၤကွၢ်အစၢတဖဉ် SD BIOLINE

	Pf	***Pv***	**Neg**	**Invalid**	**Total**
**<5 years**					
**5 to 15 years**					
**Older than 15 years (>15)**					
**Total**					

Table 3: Number of patients **TREATED** for malaria. ပှၤဆါလၢအဒိးန့ၢ်တၢ်ညဉ်ဂိၢ်ကသံဉ်နိဉ်ဂံၢ်

	Pf		***Pv***		**Total**
	Male	Female	Male	Female	
**<5 years**					
**5 to 15 years**					
**Older than 15 years (>15)**					
**Total**					

A. **Severe cases** within reporting week: ပှၤဆါလၢအနးဖဲတနွံအံၤတၢ်ပာ်ဖျါ |______|cases

B. **Death due to Malaria or suspected of Malaria** within reporting week: |______|cases
ပှၤဆါသံလၢတၢ်ညဉ်ဂိၢ်(မ့)ဆိမိဉ်လၢတၢ်ညါဂိၢ်အဃိဖဲတနွံအံၤတၢ်ပာ်ဖျါ း

C. Total pregnant women within reporting week: Neg |____| Pf |____| Pv|____|
ပှၤမိၢ်ဒၢနိဉ်ဂံၢ်လၢတနွံအံၤတၢ်ပာ်ဖျါ

Remaining number of SD BIOLINE tests in stock: တၢ်မၤကွၢ် SD BIOLINE လၢအအိဉ်တ့ၢ် |______|

Remaining number of COARTEM boxes for Pf treatment: ကသံဉ်လၢတၢ်ညဉ်ဂိၢ်ဃၢ် Pfအဂီၢ်အိဉ်တ့ၢ်|______________|
boxes

တၢ်ဂ့ၢ်တၢ်ကျိၤအဂုၤအဂၤတဖဉ်အဂီၢ်ကွဲးအီၤလၢအချၢတကပၤ.
