# Supplementary material for: Scale up of a Plasmodium falciparum elimination program and surveillance system in Kayin State, Myanmar
Source: Wellcome Open Res. 2017 Dec 22;2:98. Originally published 2017 Oct 9. [Version 2] doi: 10.12688/wellcomeopenres.12741.2 (PMC5701446; doi:10.12688/wellcomeopenres.12741.2)
Supplement: Supplementary file 3 [file wellcomeopenres-2-14723-s0002.tgz › c3a1b426-5abb-4b94-a405-cd2d2a15ed43.pdf]

Malaria Post Assessment

Village Name(သဝီမံၤ)____________________Township(ကီၢ်ဆၣ်) ______________District(ကီၢ်ရှၣ်) _________State(ကီၢ်စဲၣ်)______
[1]Malaria Post Code____________________ HH number[2]: _______________

Village GPS coordinates: LAT:__________________LONG:_____________________

Name of [3]Malaria post worker (တၢ်ညၣ်ဂံၢ်ကသံၣ်ဲၤပှၤမၤတၢ်ဖိအမံၤ)	(1) ___________________________	(2) ____________________________
TRAINING :<br><br>METF RETRAINING:	METF (3d/5d)<br>from MP supervisor<br>other/no training<br>Yes No	METF (3d/5d)<br>from MP supervisor<br>other/no training<br>Yes No
Name of MP Supervisor (တၢ်ညၣ်ဂံၢ်ကသံၣ်ဲၤအပှၤဟ့ၣ်ကူၣ်တၢ်အမံၤ) _____________________________________		
MP worker not present	Number of days since MPW away: ……… Number of days until back: ……….	
If not at post, where did the MPW go ?	…………………………………………………	

Assessment questions to MP workers (Ask to malaria workers directly) (တၢ်သံကွၢ်လၢပှၤကွၢ်တၢ်ညၣ်ဂံၢ်ဃၢ်အပှၤမၤတၢ်ဖိအဂီၢ်)

		Condition	Comment/remark
1	Was the MP closed for > 24 hours in last 2 months?<br>ဖဲအပူၤကွံာ်၂လါန့ၣ်မ့ၢ်တၢ်ကွၢ်တၢ်ညၣ်ဂံၢ်ကသံၣ်ဲၤပတုာ်ဃာ်အသးအါန့ၢ်၂၄နၣ်ရံၢ်ဧါ<br><br>If MPW was available even if MP was closed, mention in remark	YES NO	
2	Are there valid ACTs in the MP?<br>လၢတၢ်ညၣ်ဂံၢ်ကသံၣ်ဲၤပူၤန့ၣ်ကသံၣ်ယါဘျါတၢ်ညၣ်ဂံၢ်([4]ACTs)<br>လၢအသီတတလၢဘၣ်(အမုၢ်နံၤတလာ်ဘၣ်)တဖၣ်အိၣ်ဧါ	YES NO	
3	Are there vaild RDTs in the MP ?<br>လၢတၢ်ကွၢ်တၢ်ညၣ်ဂံၢ်ကသံၣ်ဲၤန့ၣ်တၢ်မၤကွၢ်တၢ်ညၣ်ဂံၢ်ဃၢ်([5]RDTs)<br>လၢအသီတတလၢဘၣ်(အမုၢ်နံၤတလာ်ဘၣ်)တဖၣ်အိၣ်ဧါ	YES NO	
4	Were there >2 days out of stocks (RDTs or ACTs) in the past 4 weeks?<br>ဖဲအပူၤကွံာ်၄နွံန့ၣ်(RDTs)မ့တမ့ၢ်(ACTs)လာ်ကွံာ်တအိၣ်လၢၤဘၣ်အါန့ၢ်ဘၣ်၂သီအိၣ်ဧါ	YES NO	If yes, ask why<br>မ့ၢ်တအိၣ်ဘၣ်န့ၣ်သံကွၢ်အီၤ<br>"ဘၣ်မနုၤအဃိလဲၣ်"
5	How are the results reported?<br>နဆှၢထီၣ်က့ၤတၢ်အစၢတဖၣ်လဲၣ်(အကျိၤအကျဲ)<br>SMS (ဆှၢခီဖျိလီတဲစိ Paper (လံာ်ကဘျံးပၤ) Other (………………)		
6	Does the MPW receive regular financial incentive?<br>တၢ်ညၣ်ဂံၢ်ဲၤပှၤမၤတၢ်ဖိတဖၣ်ဒီးန့ၢ်ဘၣ်ကျိၣ်စ့တၢ်မၤစၢၤထီဘိစ့ၢ်ကီးဧါ	YES NO	
7	Is there another MP in the village?<br>လၢသဝီပူၤအံၤမ့ၢ်တၢ်ကွၢ်တၢ်ညၣ်ဂံၢ်ဲၤအဂၤ(လၢအတမ့ၢ်နတၢ်ကရၢကရိ)ဘၣ်န့ၣ်အိၣ်ဧါ	YES NO	
8	If YES, specify the supporting organization<br>တၢ်ညၣ်ဂံၢ်ကသံၣ်ဲၤအဂုၤအဂၤမ့ၢ်အိၣ်ဘၣ်ဖုးန့ၣ်ကွဲးဖျါထီၣ်တၢ်ကရိလၢအမၤစၢၤအီၤအမံၤ	______________________________	
8b	: If Yes, do you receive malaria data from them?	YES NO	

---

[1] Malaria Post = တၢ်ညၣ်ဂံၢ်ကသံၣ်ဲၤ(တၢ်လီၢ်ဖဲပှၤလဲၤမၤကွၢ်တၢ်ညၣ်ဂံၢ်ဃၢ်,ပှၤဖိးစုနၢခိၣ်ဒီးမၤကွၢ်တၢ်ညၣ်ဂံၢ်ဃၢ်အလီၢ်)

[2] House that are inhabitated = ဲၤလၢပှၤအိၣ်

[3] Malaria Post Worker = တၢ်ညၣ်ဂံၢ်ကသံၣ်ဲၤအပှၤမၤတၢ်(ပှၤမၤတၢ်ဖိလၢအအိၣ်လၢပှၤဖိးစုနၢခိၣ်ဒီးမၤကွၢ်တၢ်ညၣ်ဂံၢ်ဃၢ်အလီၢ်)

[4] ACT = ကသံၣ်လၢတၢ်ကူစါတၢ်ညၣ်ဂံၢ်ဃၢ်

[5] RDT = Rapid Diagnosis Test

9	How often did you receive the visit of your MP supervisor in your MP in the last 2 months?<br>လၢတလါအတီၢ်ပူၤန့ၣ်,န(supervisor)ဟဲထံၣ်လိာ်အသးဒီးနၤပှဲၤဘျီလဲၣ်	…………………. time(s)
10	How often do you meet your MP supervisor outside your MP in the last 2 months?	…………………. time(s)

Assessment by Evaluator (Check - List)

1	Is there a Malaria Post Manual in the MP?<br>လံာ်နဲၣ်ကျဲလၢတၢ်ညၣ်ဂီၢ်ကသံၣ်ဒၢးအပှၤမၤတၢ်ဖိအဂီၢ်အိၣ်ဧါ	YES	NO
2	Are there reporting forms in the MP?<br>လၢတၢ်ညၣ်ဂီၢ်ကသံၣ်ဒၢးန့ၣ်တၢ်ပာ်ဖျါထီၣ်ကွၤအလံာ်တိၤဃ့(forms)အိၣ်ဧါ	YES	NO
3	Is there a logbook (daily recording of individual patients) in the MP?<br>လၢတၢ်ညၣ်ဂီၢ်ကသံၣ်ဒၢးန့ၣ်အလံာ်တၢ်မၤနီၣ်မၤဃါ(တနံၤဘၣ်တနံၤတၢ်ကွဲးနီၣ်ပှၤဆါအစရီ)အိၣ်စ့ၢ်ကီးဧါ	YES	NO
4	Are the "Days of fever" recorded for each patient? (Review the daily record sheets)<br>လၢတၢ်ကွဲးနီၣ်အပူၤန့ၣ်မ့ၢ်တၢ်ကွဲးနီၣ်မၤနီၣ်ပှၤဆါတဂၤစုာ်စုာ်အတၢ်ကိၢ်ထီၣ်ယံာ်ပှဲၤသီလဲၣ်အိၣ်စ့ၢ်ကီးဧါ<br>(ကွၢ်လၢတနံၤဘၣ်တနံၤအတၢ်ကွဲးနီၣ်အလံာ်ကွ့ပူၤ)	YES	NO
5	Are there more than 5 consecutive days without activity[6] in the logbook?<br>လၢတၢ်ကွဲးနီၣ်အလံာ်ပူၤန့ၣ်တၢ်ကွဲးနီၣ်ကွဲးဃါလီၤဟိ(တၢ်ဟူးတၢ်ဂဲၤတအိၣ်)အါန့ၢ်ဒီး၃သီအိၣ်ဧါ	YES	NO
6	Adequate or sufficient medication and supplies(observe and check carefully)<br>ကသံၣ်ကသီတၢ်ပီးတၢ်လီအိၣ်လၢအိၣ်ပှဲၤ(ကွၢ်သမံသမိးလီၤတံၢ်လီၤဆဲး)<br>SD biolines = tests ACTs= tabs<br>CQ = tabs PMQ = tabs<br>Quinine/Clindamycin =		

1. Comment or suggestions from malaria post worker.
   တၢ်ကွၢ်တၢ်ညၣ်ဂီၢ်ကသံၣ်ဒၢးအပှၤမၤတၢ်ဖိတဖၣ်အတၢ်ထံၣ်(မ့တမ့ၢ်)တၢ်ဟ့ၣ်ကူၣ်ဟ့ၣ်ဖး

   ____
   ____
   ____
   ____

2. Comments or suggestion from the observer.
   ပှၤလဲၤလီၤသမံသမိးတၢ်အတၢ်ထံၣ်(မ့တမ့ၢ်)တၢ်ဟ့ၣ်ကူၣ်ဟ့ၣ်ဖး

   ____
   ____
   ____
   ____

Name (ပှၤသမံသမိးတၢ်အမံၤ) : ____________________________

Signature (ပှၤသမံသမိးတၢ်ဆဲးလီၤစုမ့ၢ်ကျၢ်) : ______________________

Date (မုၢ်နံၤမုၢ်သီ) : __________________________

[6] Activity = (case of fever) တၢ်ဟူးတၢ်ဂဲၤ(ပှၤလီၤကိၢ်ဟဲဒိးနဲၣ်အသးအနီၣ်ဂံၢ်)
