# Supplementary material for: Scale up of a Plasmodium falciparum elimination program and surveillance system in Kayin State, Myanmar
Source: Wellcome Open Res. 2017 Dec 22;2:98. Originally published 2017 Oct 9. [Version 2] doi: 10.12688/wellcomeopenres.12741.2 (PMC5701446; doi:10.12688/wellcomeopenres.12741.2)
Supplement: Supplementary file 4 [file wellcomeopenres-2-14723-s0004.tgz › 1a5c1a17-3b97-425c-8917-93d85abd8a7a.docx]

**Supplementary File 4**: Table for baseline qPCR survey sample size calculation

| **Number of households** | **Target number of samples** |
| --- | --- |
| 5 and less | 20 |
| 6-8 | 26 |
| 9-10 | 30 |
| 11 | 31 |
| 12 | 33 |
| 13 | 34 |
| 14 | 35 |
| 15 | 36 |
| 16 | 37 |
| 17 | 38 |
| 18 | 39 |
| 19 | 40 |
| 20 | 41 |
| 21 | 42 |
| 22 | 42 |
| 23 | 43 |
| 24 | 44 |
| 25 | 44 |
| 26 | 45 |
| 27 | 45 |
| 28 | 46 |
| 29 | 46 |
| 30-34 | 48 |
| 35-39 | 49 |
| 40-44 | 51 |
| 45-49 | 52 |
| 50-59 | 54 |
| 60-69 | 56 |
| 70-79 | 57 |
| 80-89 | 57 |
| 90-99 | 58 |
| 100-124 | 59 |
| 125-149 | 59 |
| 150-199 | 61 |
| 200-499 | 63 |
| 500 and more | 65 |

Assumptions

5.5 inhabitants/household

40% malaria prevalence by qPCR (including asymptomatic + submicroscopic infections)

+/- 10% precision for a 90% binomial confidence interval

80% power
