# Supplementary material for: Scale up of a Plasmodium falciparum elimination program and surveillance system in Kayin State, Myanmar
Source: Wellcome Open Res. 2017 Dec 22;2:98. Originally published 2017 Oct 9. [Version 2] doi: 10.12688/wellcomeopenres.12741.2 (PMC5701446; doi:10.12688/wellcomeopenres.12741.2)
Supplement: Supplementary file 5 [file wellcomeopenres-2-14723-s0005.tgz › 5a611156-761c-4e30-8a08-03a9ec05e76e.docx]

**Supplementary File 5**: Table for M12 qPCR survey sample size calculation

| **Expected M12 prevalence** | **Population** | **Range for target number of samples** |
| --- | --- | --- |
| <2% | <80 | 30-32 |
| <2% | 80-90 | 35-42 |
| <2% | 90-110 | 43-49 |
| <2% | 110-150 | 54-76 |
| <2% | 150-200 | 71-80 |
| <2% | 200-250 | 92-103 |
| <2% | 250-300 | 122-143 |
| <2% | 300-500 | 135-200 |
| >2% | 90-110 | 45-50 |
| >2% | 110-150 | 50-68 |
| >2% | 150-200 | 65-90 |
| >2% | 250-300 | 100-120 |
| >2% | 250-300 | 120-145 |
| >2% | 300-500 | 136-150 |

Assumptions

95% binomial confidence interval

90% reduction from baseline P. falciparum qPCR prevalence

50% adult population (census obtained from MDA data)

precision

- if expected prevalence<2%: +/-100% of expected value

- if expected prevalence>2%: +/-50% of expected value
