# Supplementary material for: Scale up of a Plasmodium falciparum elimination program and surveillance system in Kayin State, Myanmar
Source: Wellcome Open Res. 2017 Dec 22;2:98. Originally published 2017 Oct 9. [Version 2] doi: 10.12688/wellcomeopenres.12741.2 (PMC5701446; doi:10.12688/wellcomeopenres.12741.2)
Supplement: Supplementary file 6 [file wellcomeopenres-2-14723-s0003.tgz › a9b56a83-dc24-49b4-a6d7-1b9d34503306.zip!/Supplementary File 4/Supplementary File 4a.pdf]

Household number: ____

MDA Log Book

	IDENTIFIERS						MONTH 0				MONTH 1				MONTH 2		
SR	HH	PID	NAME	Age	Sex	WT	D0	D1	D2	WT	D0	D1	D2	WT	D0	D1	D2
							Date: __ __/__ __/__ __<br>DP:__:__ __ t / ml<br>PQ __:__ __ t / ml<br>V: Y / N R: Y / N<br>S: Y / N	Date: __ __/__ __/__ __<br>DP:__:__ __ t / ml<br>V: Y / N R: Y / N<br>S: Y / N	Date: __ __/__ __/__ __<br>DP:__:__ __ t / ml<br>V: Y / N R: Y / N<br>S: Y / N		Date: __ __/__ __/__ __<br>DP:__:__ __ t / ml<br>PQ __:__ __ t / ml<br>V: Y / N R: Y / N<br>S: Y / N	Date: __ __/__ __/__ __<br>DP:__:__ __ t / ml<br>V: Y / N R: Y / N<br>S: Y / N	Date: __ __/__ __/__ __<br>DP:__:__ __ t / ml<br>V: Y / N R: Y / N<br>S: Y / N		Date: __ __/__ __/__ __<br>DP:__:__ __ t / ml<br>PQ __:__ __ t / ml<br>V: Y / N R: Y / N<br>S: Y / N	Date: __ __/__ __/__ __<br>DP:__:__ __ t / ml<br>V: Y / N R: Y / N<br>S: Y / N	Date: __ __/__ __/__ __<br>DP:__:__ __ t / ml<br>V: Y / N R: Y / N<br>S: Y / N
			Remark:				Q1. ___ Q2. ___ Q3. ___<br>Q4.1 __ Q4.2. __ Q5. ___<br>If (9) ______________	Stop participating M0? Y / N<br>Why stopped? Away Side effect Allergy<br>Other______________________________			Q1. ___ Q2. ___ Q3. ___<br>Q4.1 __ Q4.2. __ Q5. ___<br>If (9) ______________	Stop participating M1? Y / N<br>Why stopped? Away Side effect Allergy<br>Other______________________________			Q1. ___ Q2. ___ Q3. ___<br>Q4.1 __ Q4.2. __ Q5. ___<br>If (9) ______________	Stop participating M2? Y / N<br>Why stopped? Away Side effect Allergy<br>Other______________________________	
							Date: __ __/__ __/__ __<br>DP:__:__ __ t / ml<br>PQ __:__ __ t / ml<br>V: Y / N R: Y / N<br>S: Y / N	Date: __ __/__ __/__ __<br>DP:__:__ __ t / ml<br>V: Y / N R: Y / N<br>S: Y / N	Date: __ __/__ __/__ __<br>DP:__:__ __ t / ml<br>V: Y / N R: Y / N<br>S: Y / N		Date: __ __/__ __/__ __<br>DP:__:__ __ t / ml<br>PQ __:__ __ t / ml<br>V: Y / N R: Y / N<br>S: Y / N	Date: __ __/__ __/__ __<br>DP:__:__ __ t / ml<br>V: Y / N R: Y / N<br>S: Y / N	Date: __ __/__ __/__ __<br>DP:__:__ __ t / ml<br>V: Y / N R: Y / N<br>S: Y / N		Date: __ __/__ __/__ __<br>DP:__:__ __ t / ml<br>PQ __:__ __ t / ml<br>V: Y / N R: Y / N<br>S: Y / N	Date: __ __/__ __/__ __<br>DP:__:__ __ t / ml<br>V: Y / N R: Y / N<br>S: Y / N	Date: __ __/__ __/__ __<br>DP:__:__ __ t / ml<br>V: Y / N R: Y / N<br>S: Y / N
			Remark:				Q1. ___ Q2. ___ Q3. ___<br>Q4.1 __ Q4.2. __ Q5. ___<br>If (9) ______________	Stop participating M0? Y / N<br>Why stopped? Away Side effect Allergy<br>Other______________________________			Q1. ___ Q2. ___ Q3. ___<br>Q4.1 __ Q4.2. __ Q5. ___<br>If (9) ______________	Stop participating M1? Y / N<br>Why stopped? Away Side effect Allergy<br>Other______________________________			Q1. ___ Q2. ___ Q3. ___<br>Q4.1 __ Q4.2. __ Q5. ___<br>If (9) ______________	Stop participating M2? Y / N<br>Why stopped? Away Side effect Allergy<br>Other______________________________	
							Date: __ __/__ __/__ __<br>DP:__:__ __ t / ml<br>PQ __:__ __ t / ml<br>V: Y / N R: Y / N<br>S: Y / N	Date: __ __/__ __/__ __<br>DP:__:__ __ t / ml<br>V: Y / N R: Y / N<br>S: Y / N	Date: __ __/__ __/__ __<br>DP:__:__ __ t / ml<br>V: Y / N R: Y / N<br>S: Y / N		Date: __ __/__ __/__ __<br>DP:__:__ __ t / ml<br>PQ __:__ __ t / ml<br>V: Y / N R: Y / N<br>S: Y / N	Date: __ __/__ __/__ __<br>DP:__:__ __ t / ml<br>V: Y / N R: Y / N<br>S: Y / N	Date: __ __/__ __/__ __<br>DP:__:__ __ t / ml<br>V: Y / N R: Y / N<br>S: Y / N		Date: __ __/__ __/__ __<br>DP:__:__ __ t / ml<br>PQ __:__ __ t / ml<br>V: Y / N R: Y / N<br>S: Y / N	Date: __ __/__ __/__ __<br>DP:__:__ __ t / ml<br>V: Y / N R: Y / N<br>S: Y / N	Date: __ __/__ __/__ __<br>DP:__:__ __ t / ml<br>V: Y / N R: Y / N<br>S: Y / N
			Remark:				Q1. ___ Q2. ___ Q3. ___<br>Q4.1 __ Q4.2. __ Q5. ___<br>If (9) ______________	Stop participating M0? Y / N<br>Why stopped? Away Side effect Allergy<br>Other______________________________			Q1. ___ Q2. ___ Q3. ___<br>Q4.1 __ Q4.2. __ Q5. ___<br>If (9) ______________	Stop participating M1? Y / N<br>Why stopped? Away Side effect Allergy<br>Other______________________________			Q1. ___ Q2. ___ Q3. ___<br>Q4.1 __ Q4.2. __ Q5. ___<br>If (9) ______________	Stop participating M2? Y / N<br>Why stopped? Away Side effect Allergy<br>Other______________________________	
							Date: __ __/__ __/__ __<br>DP:__:__ __ t / ml<br>PQ __:__ __ t / ml<br>V: Y / N R: Y / N<br>S: Y / N	Date: __ __/__ __/__ __<br>DP:__:__ __ t / ml<br>V: Y / N R: Y / N<br>S: Y / N	Date: __ __/__ __/__ __<br>DP:__:__ __ t / ml<br>V: Y / N R: Y / N<br>S: Y / N		Date: __ __/__ __/__ __<br>DP:__:__ __ t / ml<br>PQ __:__ __ t / ml<br>V: Y / N R: Y / N<br>S: Y / N	Date: __ __/__ __/__ __<br>DP:__:__ __ t / ml<br>V: Y / N R: Y / N<br>S: Y / N	Date: __ __/__ __/__ __<br>DP:__:__ __ t / ml<br>V: Y / N R: Y / N<br>S: Y / N		Date: __ __/__ __/__ __<br>DP:__:__ __ t / ml<br>PQ __:__ __ t / ml<br>V: Y / N R: Y / N<br>S: Y / N	Date: __ __/__ __/__ __<br>DP:__:__ __ t / ml<br>V: Y / N R: Y / N<br>S: Y / N	Date: __ __/__ __/__ __<br>DP:__:__ __ t / ml<br>V: Y / N R: Y / N<br>S: Y / N
			Remark:				Q1. ___ Q2. ___ Q3. ___<br>Q4.1 __ Q4.2. __ Q5. ___<br>If (9) ______________	Stop participating M0? Y / N<br>Why stopped? Away Side effect Allergy<br>Other______________________________			Q1. ___ Q2. ___ Q3. ___<br>Q4.1 __ Q4.2. __ Q5. ___<br>If (9) ______________	Stop participating M1? Y / N<br>Why stopped? Away Side effect Allergy<br>Other______________________________			Q1. ___ Q2. ___ Q3. ___<br>Q4.1 __ Q4.2. __ Q5. ___<br>If (9) ______________	Stop participating M2? Y / N<br>Why stopped? Away Side effect Allergy<br>Other______________________________	

t= tablets
ml= ml liquid

V= Vomit R= Repeated dose S= Supervised treatment
Y= Yes N= No
