# Supplementary material for: Scale up of a Plasmodium falciparum elimination program and surveillance system in Kayin State, Myanmar
Source: Wellcome Open Res. 2017 Dec 22;2:98. Originally published 2017 Oct 9. [Version 2] doi: 10.12688/wellcomeopenres.12741.2 (PMC5701446; doi:10.12688/wellcomeopenres.12741.2)
Supplement: Supplementary file 6 [file wellcomeopenres-2-14723-s0003.tgz › a9b56a83-dc24-49b4-a6d7-1b9d34503306.zip!/Supplementary File 4/Supplementary File 4b.pdf]

METF « Non-Participant » RECORD FORM (version 1.4 – 20 Jul 2015)

Q1. WHY NOT PARTICIPATED IN MDA?

1. Stayed at home *(go to Q2)* 2. Refused *(Q3)* 3. Absent *(Q4)* 4. Not meet inclusion criteria *(Q5)*

8. Don't know *(stop)* 9. Other reason *(if checked, specify)* ……………………………………………………………………………

Q2. IF STAYED AT HOME, WHY?

1. Not feel involved
2. Not see malaria as a problem
3. Shy
4. Too busy
8. Don't know
9. Other *(if checked, specify)* ……………………………………………………………………………………………………

Q3. IF REFUSED, WHY?

1. Afraid of needles
2. Afraid of medicine
3. Afraid of side effects
4. Chronic disease
5. Experienced side effect with previous MDA, not want to take again
6. Pregnant woman (2nd or 3rd trim) or post-partum
8. Don't know
9. Other *(if checked, specify)* ……………………………………………………………………………………………………….

Q4.1 IF ABSENT:
WHERE DID THIS PERSON GO?

1. Farm
2. Forest (hunting, logging, fishing, charcoal…)
3. School/Student
4. A village nearby
5. In another village/district/township/country
8. Don't know
9. Other *(if checked, specify)* ……………………………………………………………………………………………………….

Q4.2 WHEN WILL THIS PERSON COME BACK?

1. Today
2. Will come back in less than one week
3. Will come back next month
4. Stay there for seasonal work, come back after
5. Moved permanently (only come back occasionally, holidays, visits…)
8. Don't know
9. Other *(if checked, specify)* ……………………………………………………………………………………………………….

Q5. IF NOT MEETING INCLUSION CRITERIA, WHICH

1. Child less than 1 year
2. Pregnant woman (1st trimester)
3. Allergy to anti-malaria medication
4. Unknown allergy
9. Other *(if checked, specify)* ……………………………………………………………………………………………………….
