# Supplementary material for: Scale up of a Plasmodium falciparum elimination program and surveillance system in Kayin State, Myanmar
Source: Wellcome Open Res. 2017 Dec 22;2:98. Originally published 2017 Oct 9. [Version 2] doi: 10.12688/wellcomeopenres.12741.2 (PMC5701446; doi:10.12688/wellcomeopenres.12741.2)
Supplement: Peer review history

# Peer review: Referee response for version 1

**DOI:** 10.21956/wellcomeopenres.13804.r26890

**Published:** 2017-11-22

**Authors:** Christopher V. Plowe

**Affiliations:** 1 Division of Malaria Research, Institute for Global Health, University of Maryland School of Medicine, Baltimore, MD, USA

This is a well written and thorough description of a hybrid research/programmatic activity aiming to evaluate and scale up surveillance and mass drug administration to eliminate malaria from a large area of Myanmar's Karen State encompassing hundreds of villages. The approaches and procedures are well described, with the exception that the actual sample sizes for prevalence surveys are not provided along with the description of the procedure for determining sample size. The supplemental materials may be helpful to other groups undertaking malaria elimination interventions in other areas not covered by strong health systems.

I have read this submission. I believe that I have an appropriate level of expertise to confirm that it is of an acceptable scientific standard.

# Response

**Published:** 2017-12-18

**Authors:** Daniel Parker

**Affiliations:** University of California, Irvine, USA

We thank the reviewer for their comments and suggestions.

 The reviewer is correct that this information is missing and that it could be valuable for readers. We have now included the tables (Supplementary File 4; Supplementary File 5) that were constructed to estimate survey sample sizes necessary for a given village size, based on power analyses and operational constraints (these surveys were operationally challenging because of the need for a cold chain and transporting the samples to the laboratory within 24 hours). The sample sizes for baseline surveys were smaller than follow up survey sample sizes.  Follow up surveys that were done > 12 months after MDA had larger sample sizes in order to have greater confidence (and smaller confidence intervals) post-MDA.                         

 Our goal in writing this protocol was to describe in detail (with no word limits) how the operational components of the project were set up. A subsequent manuscript describing the results of the project, including operational results, will soon follow. We would prefer to not include any specific operational or research results at this time.

# Peer review: Referee response for version 1

**DOI:** 10.21956/wellcomeopenres.13804.r26885

**Published:** 2017-10-23

**Authors:** Philip Bejon

**Affiliations:** 1 Wellcome Trust Research Programme, Kenyan Medical Research Institute (KEMRI), Kilifi, Kenya

Drug-resistant falciparum parasites are a major global emergency, hence elimination in the Myanmar/Thai border as well as the Cambodia/Thai border is highly desirable, and the project described here is a logical and important progression of the small-scale pilots carried out earlier.  Making this protocol openly available is a useful step, and the protocol is well described.  I have a number of minor comments and questions for clarification.

 The abstract discussion is written in terms of a project that has illustrated an outcome and that relied on strong rapport.  The results or evaluation of the project aren’t presented here, so perhaps this should be rephrased in terms of intention to demonstrate or likely outcomes in future tense rather than past tense.

 The introduction could provide useful additional context if it mentioned what is being done on the Thai/Cambodia border, and also the recent findings of spread of resistant parasite clones in Vietnam.

 The methods mention GPS and GLONASS.  Why were both used?  And what was done when there were discrepant readings?

 Under Community Engagement “MPs” is first used without spelling out (defined next paragraph as Malaria Posts).

 What was done when the initial CE team contact suggested concerns or frank resistance?  Were there pre-prepared FAQ sheets and other sensitization materials ready?  Were any materials translated/backtranslated?

 Were there processes established for monitoring completeness of data collection in real time and sending queries for out-of-range values?

 Malaria Prevalence Surveys: What was the software and algorithm used for the randomization process?

 How was randomization applied to villagers?  Were they all enumerated first and then randomly selected to receive a return visit or was the process more “on the ground”.  Were whole households sampled (convenient) or were villagers sampled across households?

 For the PCR how were the standards produced and quality controlled?  (Presumably from known high parasitaemia cultures diluted through uninfected blood, but this isn't stated).

 Antimalarial resistance monitoring was done at MPs but not in community surveys.  Is it possible a reservoir of resistance among low density parasitaemias in the community would be missed like this?

 Under statistical analysis could consider using SATScan, and perhaps looking at prevalence of resistance for spatial clustering as well as prevalence of infection?  For positive vs negative results it may be appropriate to look at Ripley’s K function for cases/controls rather than Moran’s I which is for continuous data, since the binned prevalences won’t consider sample size in each location, and the prevalences are unlikely to be normally distributed.

 Data: The shape files and locations of villages could be considered study data, and would be useful to those who subsequently work in the field.  However the data may well be too sensitive to post open access, in which case a note should be made regarding a data governance system via which applications for data can be made.

 Discussion: The second paragraph limitations would be readily addressed by using SATScan, which would take the arbitrary definition away from the protocol and address neighbouring villages for hotspots.

 The evaluation of effectiveness of this protocol when put into practice on the ground, and the epidemiological data generated will be very interesting and important.  This is a really major and very important undertaking.

I have read this submission. I believe that I have an appropriate level of expertise to confirm that it is of an acceptable scientific standard.

# Response

**Published:** 2017-12-18

**Authors:** Daniel Parker

**Affiliations:** University of California, Irvine, USA

Drug-resistant falciparum parasites are a major global emergency, hence elimination in the Myanmar/Thai border as well as the Cambodia/Thai border is highly desirable, and the project described here is a logical and important progression of the small-scale pilots carried out earlier.  Making this protocol openly available is a useful step, and the protocol is well described.  I have a number of minor comments and questions for clarification.

 The abstract discussion is written in terms of a project that has illustrated an outcome and that relied on strong rapport.  The results or evaluation of the project aren’t presented here, so perhaps this should be rephrased in terms of intention to demonstrate or likely outcomes in future tense rather than past tense.

**Agreed. Our primary goal in this protocol paper was to describe the establishment of the project, which is still ongoing. We have therefore changed the wording in the abstract to indicate that the establishment is what was done in the past but that the project is ongoing.**

 The introduction could provide useful additional context if it mentioned what is being done on the Thai/Cambodia border, and also the recent findings of spread of resistant parasite clones in Vietnam.

**Agreed. We have now added text mentioning that there are targeted MDA studies being done in Cambodia, Laos and Vietnam. We have also added a statement and reference about the spread of resistant clones throughout the Greater Mekong Subregion.**

 The methods mention GPS and GLONASS.  Why were both used?  And what was done when there were discrepant readings?

**The satellite-enable geo-referencing devices used for this project are both GPS and GLONASS capable.  We enabled GLONASS capability solely to increase the accuracy of our readings (at the expense of the loss of a bit of battery power). From the reviewers comment we see that our wording made it appear that we were using multiple devices simultaneously when in reality we were using one device with both GLONASS and GPS capabilities. We have now changed the wording in an attempt to make this clear:**

****

**“In order to understand the settlement demography and geography of the region, the area was systematically mapped using field teams and satellite-enabled geo-referencing devices. The devices simultaneously used GPS (global position system) and GLONAS (Globalnaya navigatsionnaya sputnikovaya sistema) satellites to increase accuracy of geographical coordinates (latitude and longitude).”**

 Under Community Engagement “MPs” is first used without spelling out (defined next paragraph as Malaria Posts).

**Thank you, we have now corrected this.**

 What was done when the initial CE team contact suggested concerns or frank resistance?

**We have now added the following statement to the CE section of the manuscript:**

**“In situations where villagers presented concerns or resistance to either surveys or MDA there were further discussions with important community, village and township leaders. A few villagers voiced concerns with regard to loss of blood that were alleviated when medical experts explained that the amount of blood being taken was minimal and would not have an effect on villagers. There were also political concerns since the project signed a memorandum of understanding with the central government and since many of these communities have been involved in a longstanding civil conflict with the military. Through meetings between the CE team and villagers, the villagers came to understand that this program was not from a central government organization, but rather an outside organization that works under the auspices of all locally and nationally relevant organizations. While participation varied in some communities, ultimately no communities completely refused to participate in MDA. Some surveys (15) could not be conducted because of a lack of willing participants. In this case, clinical incidence and consultation rates were closely monitored to ensure that population continued to trust and consult the MP in case of fever.”**

 Were there pre-prepared FAQ sheets and other sensitization materials ready? 

**Yes. We have now added the following statement to the manuscript:**

**“The CE team created and distributed community engagement materials in order to sensitize and explain the project to villagers. Materials included posters and audio announcements (statements that were created by the CE team but given to village headmen to announce in periodic village announcements). METF posters, in S’kaw Karen and Burmese languages, that encourage people to visit malaria posts when they feel sick were placed in malaria posts throughout the target area.”**

 Were any materials translated/backtranslated?

**Yes. We have now added the following statement to the ethics statement in the manuscript:**

**“The project used written informed consent, translated into S’kaw Karen – the most commonly spoken native language in the target area. The forms were back-translated by native speakers at SMRU and corrected when necessary prior to use in the field. All CE team members are fluent in the language and were able to explain the forms and the project in S’kaw Karen to villagers.”**

 Were there processes established for monitoring completeness of data collection in real time and sending queries for out-of-range values?

**Yes. We have now added the following statement to the manuscript:**

**“A data evaluation algorithm was established in order to monitor data quality. Missing reports, abnormal data reporting, spikes in clinical malaria cases, or potential problems in stock inventories were checked weekly. After potential data entry errors were excluded, the malaria post supervisors in charge of any problematic MPs were contacted to assess the situation and the required response.”**

 Malaria Prevalence Surveys: What was the software and algorithm used for the randomization process?

**We have now added the following statement to the manuscript:**

**“Villages within each grid cell were then randomly selected using sampling functions in STATA v14.1 (“sample”) and R v3.4.0 (“sample()”).”**

 How was randomization applied to villagers?  Were they all enumerated first and then randomly selected to receive a return visit or was the process more “on the ground”.  Were whole households sampled (convenient) or were villagers sampled across households?

**We now attempt to better explain this process. Participant selection was done in the field, frequently in villages with no villager list. Every attempt was made to select a representative sample but in some small villages it was necessary to select multiple people from the same houses. We have expanded this comment as follows:**

**“Following CE, survey teams approached village headmen to aid in selecting villagers for possible participation in a survey. In most cases no village census was available. Survey teams took samples from adults who agreed to participate, attempting to balance samples across sex and broad age groups, until reaching the sample size needed based on the full village population (Supplementary File 4 and Supplementary File 5). This sample size represented a significant proportion of the village population. Assuming that 50% of inhabitants were older than 18, which was verified in complete census obtained during MDA interventions, the sample size often comprised between 30 and 50% (baseline) and 50-80% (M12) of adult village population (Supplementary File 5). In follow up surveys (> M12) and in very small villages (e.g. 20 houses) it was necessary to sample multiple people from the same household.”**

 For the PCR how were the standards produced and quality controlled?  (Presumably from known high parasitaemia cultures diluted through uninfected blood, but this isn't stated).

**The qPCR followed the methods described by Imwong et al (Journal of Clinical Microbiology, 2014) including the preparation of control standards using *P. falciparum* 3D7 ring-stage synchronized cultured parasites, in which the precise number of infected red blood cells per tube was obtained using fluorescence-activated cell sorting (FACS), following the protocol described by Malleret et al. (Sci Rep, 2011)**

 Antimalarial resistance monitoring was done at MPs but not in community surveys.  Is it possible a reservoir of resistance among low density parasitaemias in the community would be missed like this?

**During surveys, participants providing a venous blood sample and other village inhabitants who accepted it received a standard RDT. A significant proportion also presented symptoms and would have consulted at the MP in the coming days. DBS were collected from *P. falciparum* positive cases, and treatment was administered. This represented ~30% of all carriers detected by qPCR in the surveys, but a small fraction of the total number of clinical cases recorded across the target area.**

**The analysis is ongoing, but preliminary results suggest that strains infecting clinical cases and asymptomatic carriers do not differ significantly in terms of resistance markers.**

 Under statistical analysis could consider using SATScan, and perhaps looking at prevalence of resistance for spatial clustering as well as prevalence of infection? 

**We have now added a statement about the use of scan statistics (SatScan) for spatial analysis of prevalence, incidence, and prevalence of resistance markers.**

 For positive vs negative results it may be appropriate to look at Ripley’s K function for cases/controls rather than Moran’s I which is for continuous data, since the binned prevalences won’t consider sample size in each location, and the prevalences are unlikely to be normally distributed.

**The reviewer brings up a good point. The original version of this manuscript was vague in explaining the spatial analyses that were used. In reality, we incorporated many more spatial analytic tools than Moran’s I or LISA statistics when exploring the data (including variography; a range of global, local and focal clustering statistics; and smoothed heatmaps).**

**Our primary goal in looking for clustering was to explore patterns in village level prevalence. From both an operational and research perspective, we wanted to know if villages with similar prevalence (as estimated from our qPCR surveys) clustered in space. We would not use Moran’s I for unstandardized count data or for absence/presence (case/control) data. The prevalence estimates accounted for village size in their design (from S Table XX) and we looked for spatial autocorrelation in the resulting continuous variable.**

**It is true that these data are not normally distributed – they are right skewed, with many villages having very low prevalence and a few (hotspots) having high prevalence. Instead of relying on p-values and test statistics, we repeated the Moran’s I calculation at multiple distance bands and examined their behavior as we compared village prevalences from villages that were farther and farther away. We also used pairwise relative semivariograms (which are known to handle skewed data well) and found the same general pattern that was exhibited by the Moran’s I correlograms we constructed (with villages that are closest being most alike).**

**We have now amended the statement in the text as follows:**

**“Statistical clustering of both incidence and prevalence was assessed using a range of geostatistical and cartographic approaches. Analytic approaches for analyzing spatial autocorrelation of population standardized incidence and prevalence data included the global Moran’s *I* statistic, local indicators of spatial autocorrelation, Kuldorf’s scan statistic, variography and spatial correlograms. It was necessary to carefully interpret results and to look for congruity between approaches because of the skewed nature of the data (with many villages having low prevalence and few having high prevalence). Choropleth maps were created for incidence at the village tract level and point maps were created and used for operational needs (e.g. to design stocking or survey strategies and to assess potential gaps in health care coverage).”**

 Data: The shape files and locations of villages could be considered study data, and would be useful to those who subsequently work in the field.  However the data may well be too sensitive to post open access, in which case a note should be made regarding a data governance system via which applications for data can be made.

**The shape files and point data for villages within the region are sensitive and ultimately belong to the several different organizations and institutions who comprise the METF. Village tract shapefiles are freely available from the Myanmar Information Mapping Unit (MIMU, http://themimu.info/gis-resources). The village tract level data are shared with the Myanmar National Malaria Program**

 Discussion: The second paragraph limitations would be readily addressed by using SATScan, which would take the arbitrary definition away from the protocol and address neighbouring villages for hotspots.

**We agree that there are several other ways (including SatScan) to define hotspots. Our goal here was simply to describe what has been done. Further work will assess the size and predictability of hotspots using a range of tools and approaches. We have now added the following statement to the limitations section:**

**“Geostatistical approaches such as the use of Kulldorff’s scan statistic may be useful for defining hotspots of infections and drug resistant strains.”**

 The evaluation of effectiveness of this protocol when put into practice on the ground, and the epidemiological data generated will be very interesting and important.  This is a really major and very important undertaking.

**Thank you for your very helpful comments, critiques and suggestions.**
